# Chemical Constituents and *α*-Glucosidase Inhibitory Activities of the Leaves of *Embelia parviflora*—In Vitro and In Silico Studies

**DOI:** 10.3390/life15050680

**Published:** 2025-04-22

**Authors:** Sy Danh Thuong, Mai Thi Hoang Anh, Nguyen Van Phuong, Chu Hoang Mau, Nguyen Huu Quan, Nguyen Thanh Cong, Le Nguyen Thanh

**Affiliations:** 1 Faculty of Biology, Thai Nguyen University of Education, Thai Nguyen University, Thai Nguyen 250000, Vietnam; 2 Department of Pharmacognosy, Faculty of Pharmacognosy and Traditional Medicine, Hanoi University of Pharmacy, Hanoi 10000, Vietnam; 3 Department of Pharmacy, Dai Nam University, Hanoi 10000, Vietnam; 4 National Institute of Medicinal Materials, Ministry of Health, Hanoi 10000, Vietnam

**Keywords:** *Embelia parviflora*, triterpene, flavonoid, megastigmane, phenolic, *α*-glucosidase

## Abstract

Phytochemical investigation of the methanol extract of *Embelia parviflora* Wall. Ex A. DC. leaves (Primulaceae family) led to the isolation of sixteen compounds including three sterols (**1**–**3**), one triterpene (**4**), four flavonoids (**5**–**8**), four megastigmanes (**9**–**12**), three phenolic compounds (**13**–**15**), and one furan derivative (**16**). Their chemical structures were determined based on ESI-MS and NMR spectral data. This is the first chemical study of *E. parviflora*. Compounds **3**, **8**–**13**, and **16** were found in the *Embelia* genus for the first time. Compounds **9**–**11**, **13**, and **16** represent the first isolation from the Primulaceae family. In the *α*-glucosidase activity assay, MeOH extract, compounds **4** and **5** strongly inhibited enzyme *α*-glucosidase activity. A molecular docking study revealed that compounds **4** and **5** showed different interactions with enzyme *α*-glucosidase.

## 1. Introduction

*Embelia* Burm. f. belongs to a genus of tropical climber plants. It was previously placed in the Myrsinaceae family but is currently classified under the Primulaceae family [1]. This genus comprises around 100 species [1], many of which have been widely used in traditional medicine systems such as Ayurveda, Siddha, and Chinese medicine for centuries. Dried berries from this genus possess various biological activities, including antibacterial, antidiabetic, anthelmintic, and carminative properties [2]. Phytochemical investigations have identified diverse compounds in *Embelia* species, including triterpenoids, alkaloids, flavonoids, steroids, alkylresorcinols, and phenolics [3,4,5,6,7,8,9,10,11,12,13,14]. Pharmacological studies, which focused on *Embelia ribes* and embelin, the main compound in the fruits of *E. ribes*, further indicate that the *E. ribes* extract and isolated compounds exhibit many different biological effects, such as antidiabetic [15,16,17,18,19,20,21], anti-obesity [21,22], antipsychotic [23,24], neuroprotective [25,26,27], antioxidant, antimicrobial, and antiviral effects [28,29,30]. In addition, *E. ruminata* and *E. schimperi* extracts showed antioxidant, anticancer, and anthelmictic activities [31,32].

In Vietnam, fifteen *Embelia* species have been documented, but only *Embelia ribes* stems and leaves were investigated for the chemical constituents and *α*-glucosidase inhibitory activity [8,9]. *Embelia parviflora* Wall. Ex A. DC. is a climbing shrub sparsely distributed in the forests of Malaysia, India, China, and Vietnam. It has been traditionally used to promote circulation, alleviate pain, and treat gynecological disorders such as irregular menstruation and amenorrhea [33]. In the northern parts of Vietnam, *E. parviflora* is known as ‘Thien Ly Huong’ and has been used as a traditional medicine for the treatment of diabetes, inflammation, oral and throat troubles, and skin diseases [34]. To date, research on the phytochemical constituents and biological activities of *E. parviflora* remains limited. The essential oil composition of *E. parviflora* has been analyzed, revealing 11 constituents in the root (77.67% of total volatile oil), 36 in the stems (92.88%), and 74 in the leaves (85.11%) [35]. Notably, *E. parviflora* has exhibited antioxidant [36] and anti-inflammatory [36] activities, as well as potential hematopoietic effects [37]. In our screening results, the MeOH extract showed 84% inhibition of *α*-glucosidase enzyme activity at the concentration of 256 μg/mL. Further research is needed to fully elucidate its phytochemical profile and pharmacological properties. As part of our continuing search for bioactive compounds from *Embelia* species, we report here the isolation and structural elucidation of sixteen compounds from the leaves of *E. parviflora* collected in Bac Kan province, Vietnam. These include three sterols (**1**–**3**), one triterpene (**4**), four flavonoids (**5**–**8**), four megastigmane derivatives (**9**–**12**), three phenolic compounds (**13**–**15**), and one furan derivative (**16**). Their chemical structures were determined based on ESI-MS and NMR spectroscopic data. The MeOH extract and isolated compounds were evaluated for their *α*-glucosidase inhibitory activity. The interactions of active compounds with *α*-glucosidase enzyme were studied by molecular docking approach.

## 2. Materials and Methods

### 2.1. Plant Materials

The leaf samples were collected from the Bac Kan province, Vietnam, in 2021. The plant was taxonomically identified as *Embelia parviflora* Wall. Ex A. DC. by Assoc. Prof. Dr. Sy Danh Thuong, Thai Nguyen University of Education, Thai Nguyen University. A voucher specimen (Thuong18102021.01) has been deposited at the Herbarium of the Department of Biology, Thai Nguyen University of Education, Thai Nguyen University.

### 2.2. General Experimental Procedures

All chemical solvents (analytical grade) were obtained from a reputable chemical supplier and used as received without additional purification. Enzyme *α*-glucosidase from *Saccharomyces cerevisiae* (E.C. 232-604-7) and *p*-nitrophenyl-α-D-glucopyranoside were purchased from Sigma Aldrich (St. Louis, MO, USA). Dimethyl sulfoxide and acarbose were obtained from Merck (Darmstadt, Germany). For column chromatography (CC), the following materials were employed: silica gel (Merck, 230–400 mesh, Darmstadt, Germany), Sephadex^®^ LH-20 (Sigma Aldrich), MCI, and Diaion HP-20 resins (Mitsubishi Chemical, Tokyo, Japan). Thin-layer chromatography (TLC) was performed using precoated aluminum silica gel plates (Merck 60 F254, Darmstadt, Germany), and spots were visualized by spraying with 10% aqueous sulfuric acid followed by heating. Nuclear magnetic resonance (NMR) spectra, including ^1^H, ^13^C, HSQC, and HMBC, were recorded on either a Bruker AVANCE 500 MHz or a Bruker AVANCE NEO 600 MHz spectrometer (Bruker, Billerica, MA, USA) at the Institute of Chemistry, the Vietnam Academy of Science and Technology, with tetramethylsilane (TMS) as the internal standard. Electrospray ionization mass spectrometry (ESI-MS) data were acquired using an Agilent 1260 series single quadrupole LC/MS system (Agilent Technologies, Palo Alto, CA, USA).

### 2.3. Extraction and Isolation

The dried leaves of *E. parviflora* (3.6 kg) were marcerated with MeOH (4 × 20 L for 24 h) at room temperature. The MeOH solvents were removed using a vacuum. The crude extract (280 g) was suspended with distilled water (1 L) and extracted consecutively with *n*-hexane and EtOAc to give *n*-hexane (46 g), EtOAc residue (96 g), and water layer, respectively. The water fraction (140 g) was applied to Diaion HP-20 CC and eluted with a solvent mixture of MeOH/water (0/100, 50/50, and 100/0, *v*/*v*) to yield a MeOH fraction (25 g).

The *n*-hexane residue (45 g) was subjected to silica gel CC (225 g, column 8 cm size) and eluted with a gradient of *n*-hexane/EtOAc (100/1–0/100, *v*/*v*) to yield 6 fractions (H1–H6). Compound **1** (*β*-sitosterol) was obtained from fraction H4 (230 mg) by crystallization.

The EtOAc extract (95 g) was chromatographed using silica gel CC (300 g, column 10 cm size) and eluted with a gradient of *n*-hexane/EtOAc (100/1–0/100, *v*/*v*) to yield 9 fractions (E1–E9). Fraction E4 (2.09 g) was subjected to silica gel CC (40 g, column 3 cm size) and eluted with *n*-hexane/EtOAc (9:1, *v*/*v*) to afford 7 fractions E4.1–E4.7. Fraction E4.2 (35 mg) was further purified by silica gel CC (7 g, column 1.5 cm size) and eluted with *n*-hexane/acetone (9:1, *v*/*v*) to give compound **4** (4.3 mg).

Fraction E6 (2.0 g) was fractionated by Sephadex^®^ LH-20 CC (90 g, column 2.5 cm size) and eluted with MeOH/CH_2_Cl_2_ (9:1, *v*/*v*) to yield 10 fractions E6.1–E6.10. Fraction E6.5 (135 mg) was purified by silica gel CC (27 g, column 2.0 cm size) and eluted with *n*-hexane/acetone (8:2, *v*/*v*) to give compound **5** (5.4 mg). Fraction E6.2 (1.3 g) was purified by Sephadex^®^ LH-20 CC (90 g, column 2.5 cm size) eluted with MeOH to yield 7 fractions E6.2.1–E6.2.7. Fraction E6.2.5 (116 mg) was purified by silica gel CC (25 g, column 2.0 cm size) and eluted with *n*-hexane/EtOAc (8:2, *v*/*v*) to give compound **3** (6.1 mg). Compound **14** (2.1 mg) was obtained from fraction E.6.2.2 (66 mg) by crystallization.

Fraction E9 (12.1 g) was chromatographed on silica gel CC (180 g, column 5 cm size) eluted with CH_2_Cl_2_/MeOH (10:1–0/1, *v*/*v*) to give 9 fractions E9.1–E9.9. Fraction E9.4 (22 mg) was further purified by silica gel CC (7 g, column 1.5 cm size) and eluted with *n*-hexane/EtOAc (7:3, *v*/*v*) to give compound **15** (2.2 mg). Compound **2** (5 mg) was obtained from fraction E9.7 (76 mg) by crystallization in CH_2_Cl_2_. Fraction E9.9 (1.7 g) was fractionated by MCI gel CC (120 g, column 3 cm size) and eluted with MeOH/H_2_O (1:1, *v*/*v*) to give compound **6** (10.2 mg) and seven fractions E.9.9.1–E.9.9.7. Fraction E9.7.5 (24 mg) was purified by Sephadex^®^ LH-20 CC (20 g, column 1.5 cm size), using MeOH as the mobile phase to afford compound **7** (12.2 mg).

The MeOH fraction (25 g) was applied to silica gel CC (150 g, column 6 cm size) and eluted with CH_2_Cl_2_/MeOH (10:1–0/1, *v*/*v*) to give 14, fractions W1–W14. Fraction W3 (180 mg) was purified by MCI gel CC (40 g, column 2 cm size) and eluted with MeOH/H_2_O (1:1, *v*/*v*) to obtain compound **16** (15.1 mg) and 9 fractions, W3.1–W3.9. Fraction W3.7 (41.5 mg) was purified by preparative HPLC and eluted with MeOH/water (40%) to yield compound **9** (4.2 mg). Fraction W3.9 (6.5 mg) was purified by preparative HPLC and eluted with MeOH/water (40%) to give compound **13** (2.1 mg). Fraction W4 (280 mg) was purified by MCI gel CC (60 g, column 2 cm size) and eluted with MeOH/H_2_O (1:2–1:0, *v*/*v*) to yield 9 fractions, W4.1–W4.9. Fraction W4.3 (22 mg) was purified by silica gel CC (7 g, column 1.5 cm size) and eluted with *n*-hexane/acetone (4:1, *v*/*v*) to give compound **10** (2.5 mg). Fraction W4.9 (22 mg) was purified by silica gel CC (10 g, column 1.5 cm size) and eluted with *n*-hexane/acetone (2:1, *v*/*v*) to yield compound **12** (2.5 mg). Fraction W12 (180 mg) was purified by reversed-phase CC (70 g, column 2.5 cm size) and eluted with MeOH/H_2_O (1:2–2:1, *v*/*v*) to afford 5 fractions, W12.1–W12.5. Fraction W12.1 (41.5 mg) was purified by preparative HPLC and eluted with acetonitrile/water (1:4, *v*/*v*) to yield compound **11** (4.2 mg). Fraction W12.3 (200 mg) was fractionated by silica gel CC (40 g, column 2 cm size) and eluted with CH_2_Cl_2_/MeOH/water (5:1:0.05, *v*/*v*/*v*) and then Sephadex^®^ LH-20 CC (50 g, column 2 cm size), using MeOH as the mobile phase to give compound **8** (10.2 mg).

### 2.4. Assay of α-Glucosidase Enzyme Inhibition

The in vitro assay of *α*-glucosidase enzyme inhibition of the *E. parviflora* MeOH extract and tested compounds was conducted following the methodology outlined in our earlier study [38]. Briefly, solutions of MeOH extract and isolated compounds at 256 μg/mL, 64 μg/mL, 16 μg/mL, 4 μg/mL, and 1 μg/mL concentrations were prepared using DMSO (Merck). The compound solution (2 μL) and 0.2 U/mL *α*-glucosidase enzyme solution (25 µL) in 120 µL phosphate buffer were mixed. After 5 min preincubation, a solution of 2.5 mM *p*-nitrophenyl *α*-D-glucopyranoside (25 µL) prepared in phosphate buffer was added. The reaction mixture was incubated at 37 °C for 30 min and was stopped by adding 0.2 M of Na_2_CO_3_ (100 µL). Enzymatic activity (the absorbance of the released *p*-nitrophenol) was quantified by measuring at 410 nm using a Biotek reader. The % inhibition was calculated using the following equation:

Inhibition (%) = [1 − (A_sample_/A_control_)] × 100

The IC_50_ value was defined as the concentration of compound that inhibited 50% of *α*-glucosidase enzyme activity and was calculated by using the program Table Curve. Acarbose, a well-known *α*-glucosidase inhibitor, served as the positive control in this experiment.

### 2.5. Molecular Docking

In this study, molecular docking was performed to investigate the mechanisms of interaction between potential compounds identified from the in vitro assay and *α*-glucosidase, following a well-established protocol [39]. Since the crystal structure of *α*-glucosidase from *Saccharomyces cerevisiae* is unavailable, a homology model was obtained from isomaltase in the RCSB Protein Data Bank (https://www.rcsb.org, accessed on 15 February 2025) (PDB ID: 3AJ7) as a template. In the next step, water molecules were removed, followed by adding hydrogen atoms and assigning partial charges. Ligand structures were downloaded from PubChem and converted into pdbqt format using Autodock Tool 1.5.6. After that, molecular docking was conducted using Autodock Vina, with a grid box size of 25 × 25 × 25 Å^3^. The grid center was set at x = 20.226, y = −8.148, and z = 17.909 for **5**–**7**, while for compound **4**, it was adjusted to x = 20.315, y = −26.388, and z = 27.802. The positive control used in this study is acarbose, and docking results were analyzed using BIOVIA Discovery Studio Visualizer 4.5.

### 2.6. Statistical Analysis

The biological experiments were performed in triplicate. The IC_50_ values are presented as the mean ± standard deviation (S.D) using the program Statistica 10.

## 3. Results and Discussion

### 3.1. Chemical Constituents and Chemotaxonomy Significance

**Figure 1:**
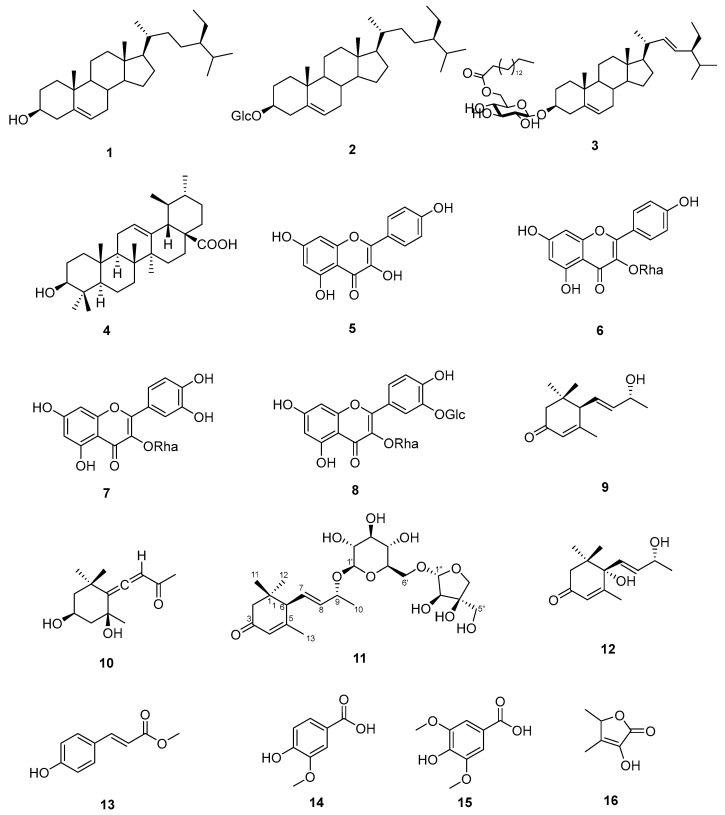
The chemical structure of isolated compounds **1**–**16** from the leaves of *E. parviflora*.

Combined chromatographic separation of the *n*-hexane, ethyl acetate, and water fractions from the MeOH extract of *E. parviflora* leaves afforded sixteen known compounds (**1**–**16**) (Figure 1) including three sterols (**1**–**3**), one triterpene (**4**), four flavonoids (**5**–**8**), four megastigmanes (**9**–**12**), three phenolic compounds (**13**–**15**), and one furano derivative (**16**). The chemical structures of the isolated compounds were identified as *β*-sistosterol (**1**) [40], daucosterol (**2**) [41], 3-*O*-(6′-*O*-palmitoyl)-*β*-D-glucopyranosyl stigmasterol (**3**) [42], ursolic acid (**4**) [43], kaempferol (**5**) [44], kaempferin (**6**) [45], quercitrin (**7**) [46], quercetin-3-rhamnoside-3′-glucoside (**8**) [47], (6*R*,9*R*)-9-hydroxy-4,7-megastigmadien-3-one (**9**) [48], grasshopper ketone (**10**) [49], (6*R*,7*E*,9*R*)-9-hydroxy-4,7-megastigmadien-3-one-9-*O*-*β*-D-apiofuranosyl(1->6)-*β*-D-glucopyranoside (**11**) [50], vomifoliol (**12**) [51], methyl *trans*-*p*-coumarate (**13**) [52], vanillic acid (**14**) [53], syringic acid (**15**) [54], and sotolone (**16**) [55,56] by the comparison of the NMR spectral data (Supplementary Materials) with those in the literature [40,41,42,43,44,45,46,47,48,49,50,51,52,53,54,55,56]. Interestingly, several pairs of an aglycone and its glycosides were found in our chemical study, such as compounds **1** and **2**, compounds **5** and **6**, and compounds **9** and **11**. In addition, two flavonoid glycosides, **7** and **8**, have the same quercetin skeleton.

To the best of our knowledge, this is the first phytochemical study of *E. parviflora* leaves. Sterols **1** and **2** are common compounds found in many plants, while compound **3** was isolated from a few plant families like Primulaceae [42,57] and Pontederiaceae [58] (Table 1 and Table 2). Sitosterol (**1**) and daucosterol (**2**) have been identified in leaf extracts of *E. ribes* and *E. rowlandii*, respectively [5]. In addition, ursolic acid (**4**), kaempferol (**5**), kaempferin (**6**), and quercitrin (**7**) have been isolated from *E. ribes* leaves [9]. Two phenolic acids, **14**–**15**, have been reported in the leaves of *E. laeta* [11] (Table 1).

In our study, compounds **3**, **8**–**13**, and **16** represent the first isolation of these compounds from the *Embelia* genus. The partial distribution in plants of compounds **3**, **8**–**13**, and **16** is presented in Table 2. Compounds **3**, **8**, and **12** were previously isolated from the Primulaceae family [42,47,57,72]. Compounds **9**–**11**, **13**, and **16** were found in the Primulaceae family for the first time.

As shown in Table 2, compounds **12** and **13** were distributed in many plant families, such as Annonaceae, Bignoniaceae, Celastraceae, Caryophyllaceae, Euphorbiaceae, Myrtaceae, Onagraceae, Rhizophoraceae, Rutaceae, Rubiaceae, Solanaceae, etc. The occurrences of megastimanes **9**–**11** in plants are quite limited. They were identified from Apocynaceae, Caprifoliaceae, Commelinaceae, Cornaceae, Cannabaceae, Chenopodiaceae, Lamiaceae, Magnoliaceae, Nelumbonaceae, Rosaceae, and Piperaceae families. As mentioned earlier, sterol **3** was isolated from the Primulaceae and Pontederiaceae families. Notably, compounds **8** and **16** were only discovered from *Myrsine seguinii* (Primulaceae) and *Quararibea funebris* (Bombacaceae), respectively. Our phytochemical study enriches our understanding of the chemical constituents of *Embelia* species and further provides a basis for the chemical taxonomic research of *E. parviflora*. Compounds **3**, **8**–**13**, and **16** might be regarded as potential fingerprint markers for *E. parviflora* plant.

### 3.2. α-Glucosidase Inhibitory Activity

The antidiabetic activity of *Embelia* species, especially *E. ribes*, has been extensively studied. In Vietnam, the *α*-glucosidase inhibitory activity of *E. ribes* stems and leaves have been reported [8,9]. Therefore, in our investigation, the *α*-glucosidase inhibitory activity of the methanol (MeOH) extract of *E. parviflora* and its isolated compounds was assessed. Acarbose, a widely recognized *α*-glucosidase inhibitor, served as the positive control, demonstrating an IC_50_ value of 198.5 ± 6.25 μg/mL. The MeOH extract showed strong inhibition of *α*-glucosidase activity with an IC_50_ of 12.80 ± 0.62 μg/mL. Compounds **4** and **5** showed strong inhibition with IC_50_ values of 1.40 ± 0.06 μg/mL and 1.75 ± 0.08 μg/mL, respectively, whereas compounds **6** and **7** exhibited moderate activity, with IC_50_ values of 162.13 ± 3.28 μg/mL and 168.01 ± 4.15 μg/mL, respectively.

This is the first report of the *α*-glucosidase inhibitory activity of compounds **3**, **8**–**11**, and **16**, but unfortunately, these isolated compounds were inactive (Table 3). Our biological results were quite similar to the results of previous reports. *β*-Sistosterol (**1**) and daucosterol (**2**) were documented to have weak anti-*α*-glucosidase activity, with IC_50_ values of 283.67 μg/mL and 247.35 μg/mL, which are close to our results [93]. Ding et al. have reported that ursolic acid (**4**) showed an enzyme inhibitory effect with an IC_50_ value of 16.9 μM (7.7 μg/mL) in a non-competitive manner [94]. In Peng’s study, kaempferol (**5**) showed strong activity against glucosidase with an IC_50_ value of 11.6 μM (3.32 μg/mL) [95]. Similar to our results, vomifoliol (**12**) was also found inactive in the biological assay [96].

However, different inhibitory effects have also been found in the literature. In the investigation of Dang’s group [9], compounds **5**–**7** showed moderate inhibitory activity, with IC_50_ values of 84.9, 94.7, and 26.5 μM, respectively. Compound **13** was reported to exhibit the inhibition of *α*-glucosidase, with an IC_50_ value of 54.15 μM [97]. Perhaps the different assay conditions have led to different results in terms of the activity.

Overall, our biological results suggest that the MeOH extract of *E. parviflora* and several isolated compounds, i.e., compounds **4**–**7**, can be used as a source for the development of natural antidiabetic agents.

### 3.3. Molecular Docking

To further investigate their interactions with the protein, molecular docking was performed. Previous research indicated that flavonoids, such as kaempferol, inhibited *α*-glucosidase through a competitive mechanism by binding to the enzyme’s active site [95,98]. In contrast, triterpenoids like ursolic acid achieve their inhibitory effect via interactions with the allosteric site [94,99]. Therefore, in this study, compounds **5**–**7** were docked into the enzyme’s active site, while **4** was docked into the allosteric site. The results are summarized in Table 4.

**Figure 2:**
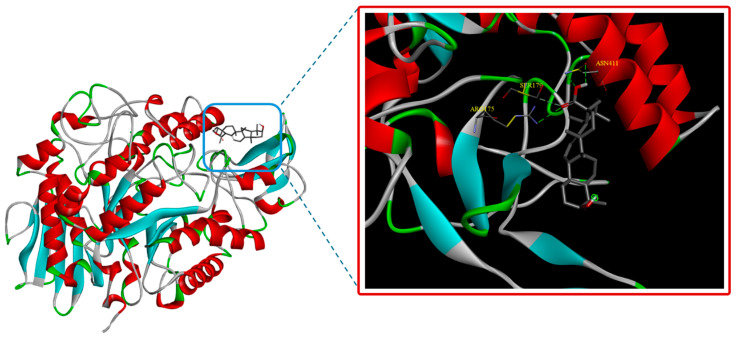
The binding mode of ursolic acid (**4**) with *α*-glucosidase enzyme.

The results showed that the positive control, acarbose, exhibited good affinity for the enzyme, with a binding energy of −6.7 kcal/mol, corresponding to an IC_50_ value of 198.5 ± 6.25 μg/mL. Notably, compound **4** demonstrated strong binding to the allosteric site of *α*-glucosidase, with a binding energy of −9.2 kcal/mol. This finding aligns well with the low IC_50_ value of ursolic acid (**4**) determined from the in vitro assay (1.40 μg/mL). Our results also indicated that ursolic acid (**4**) strongly interacts with *α*-glucosidase via hydrogen bonding and Van der Waals interactions with residues Arg175, Ser179, and Asn411 (Figure 2). Interestingly, these interactions are consistent with previous studies on the mode of binding of ursolic acid to enzymes. Ding et al. reported that ursolic acid exhibits potent *α*-glucosidase inhibitory activity through interactions with multiple enzyme residues, including Ser179 and Asn411, where hydrogen bonding plays a crucial role in its effect [94]. Similarly, a study by Elmira F. Khusnutdinova et al. on betulinic acid, a compound structurally similar to ursolic acid, found that hydrogen bonding with Arg175 and Ser179 is essential for the *α*-glucosidase inhibitory activities of this class of compounds [100].

**Figure 3:**
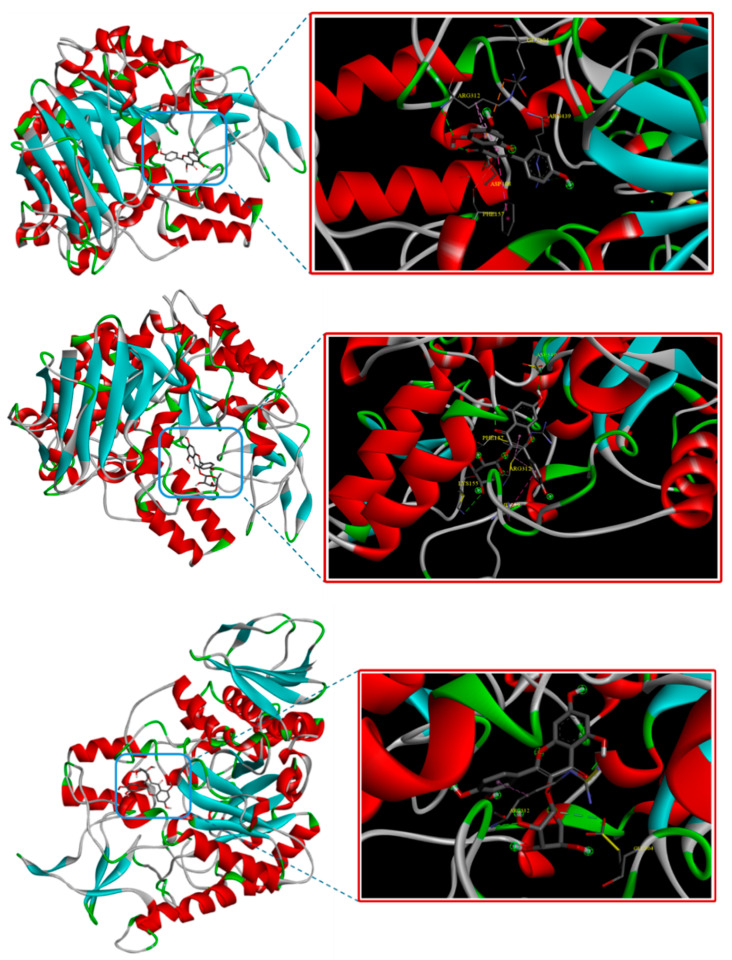
The binding modes of compounds **5**, **6**, and **7** with *α*-glucosidase enzyme.

Regarding compound **5**, the results showed that this compound could strongly bind to *α*-glucosidase, with a binding affinity of −7.9 kcal/mol. This result was consistent with a study by Xi Peng et al., in which the binding energy between kaempferol and the *α*-glucosidase was determined as −7.12 kcal/mol [95]. Further analysis revealed that **5** interacted with the enzyme through hydrogen bonds formed between its C4′-OH, C3-OH, and C5-OH groups and the residues Arg312, Asp408, and Arg439 (Figure 3).

This observation aligned well with a previous study by Nana Li et al., which demonstrated that the C4′-OH, C5′-OH, and C3-OH groups played a crucial role in the interaction between flavonoids and *α*-glucosidase enzyme [101]. In contrast, while compounds **6** and **7** exhibited strong binding affinities of −9.3 and −9.1 kcal/mol, respectively, their in vitro inhibitory activity was not as potent as that of kaempferol. This may be due to their larger molecular size, which likely hinders their access to the enzyme’s active site compared to the aglycone form. As a result, their inhibitory effect is weaker [95]. Additionally, the glycosylation of the C3-OH group may prevent these compounds from forming critical hydrogen bonds with the enzyme. Accordingly, despite their high binding affinity, their interactions may be less significant for *α*-glucosidase inhibitory activities than those of kaempferol (**5**) [101].

## 4. Conclusions

Sixteen compounds including *β*-sistosterol (**1**), daucosterol (**2**), 3-*O*-(6′-*O*-palmitoyl)-*β*-D-glucopyranosyl stigmasterol (**3**), ursolic acid (**4**), kaempferol (**5**), kaempferin (**6**), quercitrin (**7**), quercetin-3-rhamnoside-3′-glucoside (**8**), (6*R*,9*R*)-9-hydroxy-4,7-megastigmadien-3-one (**9**), grasshopper ketone (**10**), (6*R*,7*E*,9*R*)-9-hydroxy-4,7-megastigmadien-3-one-9-*O*-*β*-D-apiofuranosyl(1->6)-*β*-D-glucopyranoside (**11**), vomifoliol (**12**), methyl *p*-coumarate (**13**), vanillic acid (**14**), syringic acid (**15**), and sotolone (**16**) were isolated from the leaves of *E. parviflora*. This is the first phytochemical investigation of *E. parviflora*. Compounds **3**, **8**–**13**, and **16** were first reported in the *Embelia* genus. Compounds **9**–**11**, **13**, and **16** have not been previously discovered in the Primulaceae family. In the biological assay, the MeOH extract of *E. parviflora* leaves and isolated compounds **4** and **5** showed strong inhibition of enzyme *α*-glucosidase activity with IC_50_ values of 12.80 ± 0.62 μg/mL, 1.40 ± 0.06 μg/mL, and 1.75 ± 0.08 μg/mL, respectively. Molecular docking studies revealed that compounds **4** and **5** had different binding modes to enzyme *α*-glucosidase. Triterpene (**4**) interacts with an allosteric site of the enzyme with a binding energy of −9.2 kcal/mol, whereas flavonoid (**5**) binds to an active site of *α*-glucosidase enzyme with a binding affinity of −7.9 kcal/mol. The strong *α*-glucosidase inhibitory activity of compounds **4** and **5** made the most significant contribution to the antidiabetic activity of *E. parviflora* leaves.

## Figures and Tables

**Table 1:** The distribution of compounds **1**–**2**, **4**–**7**, and **14**–**15** in the *Embelia* plants.

Compounds	Name	Plants	Parts	References
**1**	*β*-Sistosterol	*E. ribes*	leaves	[3]
**2**	Daucosterol	*E. rowlandii*	leaves	[5]
		*E. ribes*	leaves	[3]
**4**	Ursolic acid	*E. ribes*	leaves	[9]
**5**	Kaempferol	*E. ribes*	leaves	[9]
**6**	Kaempferin	*E. ribes*	leaves	[9]
**7**	Quercitrin	*E. ribes*	leaves	[9]
**14**	Vanillic acid	*E. laeta*	leaves	[11]
**15**	Syringic acid	*E. laeta*	leaves	[11]

**Table 2:** The partial distribution of compounds **3**, **8**–**13**, and **16**.

Compounds	Species	Family	References
3-*O*-(6′-*O*-Palmitoyl)-*β*-D-glucopyranosyl stigmasterol (**3**)	*Myrsine pellucida*	Primulaceae	[42]
	*Labisia pumila*	Primulaceae	[57]
	*Monochoria vaginalis*	Pontederiaceae	[58]
Quercetin-3-rhamnoside-3′-glucoside (**8**)	*Myrsine seguinii*	Primulaceae	[47]
(6*R*,9*R*)-9-Hydroxy-4,7-megastigmadien-3-one (**9**)	*Peperomia heyneana*	Piperaceae	[59]
	*Nelumbo nucifera*	Nelumbonaceae	[60]
	*Valeriana officinalis var. latifolia*	Caprifoliaceae	[61]
	*Manglietia aromatica*	Magnoliaceae	[62]
	*Tradescantia albiflora*	Commelinaceae	[63]
Grasshopper ketone (**10**)	*Nelumbo nucifera*	Nelumbonaceae	[60]
	*Marsdenia tenacissima*	Apocynaceae	[64]
	*Anisomeles indica*	Lamiaceae	[65]
	*Humulus japonicus*	Cannabaceae	[66]
	*Chenopodium album*	Chenopodiaceae	[67]
(6*R*,7*E*,9*R*)-9-Hydroxy-4,7-megastigmadien-3-one, 9-*O*-*β*-D-Apiofuranosyl(1->6)-*β*-D-glucopyranoside (**11**)	*Eriobotrya japonica*	Rosaceae	[68]
	*Alangium premnifolium*	Cornaceae	[69]
	*Cydonia vulgaris*	Rosaceae	[70]
	*Wrightia antidysenterica*	Apocynaceae	[71]
Vomifoliol (**12**)	*Maesa membranacea*	Primulaceae	[72]
	*Physalis minima*	Solanaceae	[73]
	*Syzygium cerasiforme*	Myrtaceae	[74]
	*Rhizophora apiculata*	Rhizophoraceae	[75]
	*Palicourea adusta*	Rubiaceae	[76]
	*Euphorbia heterophylla*	Euphorbiaceae	[77]
	*Silene firma*	Caryophyllaceae	[78]
	*Epilobium angustifolium*	Onagraceae	[79]
	*Eucalyptus globulus*	Myrtaceae	[80]
	*Eucommia ulmoides*	Eucommiaceae	[81]
Methyl *trans*-*p*-coumarate (**13**)	*Zanthoxylum nitidum*	Rutaceae	[82]
	*Boehmeria virgata*	Urticaceae	[83]
	*Clausena lansium*	Rutaceae	[84]
	*Eugenia dysenterica*	Myrtaceae	[85]
	*Idesia polycarpa*	Salicaceae	[86]
	*Stereospermum acuminatissimum*	Bignoniaceae	[87]
	*Goniothalamus laoticus*	Annonaceae	[88]
	*Calocedrus formosana*	Cupressaceae	[89]
	*Tupistra chinensis*	Liliaceae	[90]
	*Annona cherimola*	Annonaceae	[91]
	*Hibiscus sabdariffa*	Malvaceae	[92]
Sotolone (**16**)	*Quararibea funebris*	Bombacaceae	[55]

**Table 3:** *α*-Glucosidase inhibitory activity of the MeOH extract of *E. parviflora* and isolated compounds.

No.	Compounds	IC_50_ (µg/mL)	No.	Compounds	IC_50_ (µg/mL)
1	**1**	>256	9	**9**	>256
2	**2**	>256	10	**10**	>256
3	**3**	>256	11	**11**	>256
4	**4**	1.40 ± 0.06	12	**12**	>256
5	**5**	1.75 ± 0.08	13	**13**	>256
6	**6**	162.13 ± 3.28	14	**16**	>256
7	**7**	168.01 ± 4.15	15	**MeOH extract**	12.80 ± 0.62
8	**8**	>256	16	**Acarbose**	198.5 ± 6.25

**Table 4:** Molecular docking results between potential compounds and *α*-glucosidase.

Compound	Binding Energy (kcal/mol)		Interacted Residues
	Active Site	Allosteric Site	
4	N.D *	−9.2	Arg175, Ser179, Asn411
5	−7.9	N.D *	Glu304, Arg312, Arg439, Asp408, Phe157
6	−9.3	N.D *	Lys155, Asp349, Phe157, Arg312, His239
7	−9.1	N.D *	Arg312, Glu304,
Acarbose	−6.7	N.D *	Glu304, His279, Pro309, Phe300, Arg312, Glu276, Gln350, Asp349, Tyr313, Asp408, Phe157

* N.D: not determined.

## Data Availability

The data presented in this study are available upon request from the corresponding author.

## Supplementary Materials

The following supporting information can be downloaded at: https://www.mdpi.com/article/10.3390/life15050680/s1, Physical and Spectroscopic Data of Compounds **1**–**16**; Figure S1. ^1^H-NMR spectrum of compound **1**; Figure S2. ^1^H-NMR spectrum of compound **2**; Figure S3. ^13^C-NMR spectrum of compound **2**; Figure S4. ^1^H-NMR spectrum of compound **3**; Figure S5. ^13^C-NMR spectrum of compound **3**; Figure S6. ^1^H-NMR spectrum of compound **4**; Figure S7. ^13^C-NMR spectrum of compound **4**; Figure S8. ^1^H-NMR spectrum of compound **5**; Figure S9. ^13^C-NMR spectrum of compound **5**; Figure S10. ^1^H-NMR spectrum of compound **6**; Figure S11. ^13^C-NMR spectrum of compound **6**; Figure S12. ^1^H-NMR spectrum of compound **7**; Figure S13. ^13^C-NMR spectrum of compound **7**; Figure S14. ^1^H-NMR spectrum of compound **8**; Figure S15. ^13^C-NMR spectrum of compound **8**; Figure S16. HSQC spectrum of compound **8**; Figure S17. HMBC spectrum of compound **8**; Figure S18. ^1^H-NMR spectrum of compound **9**; Figure S19. ^13^C-NMR spectrum of compound **9**; Figure S20. ^1^H-NMR spectrum of compound **10**; Figure S21. ^13^C-NMR spectrum of compound **10**; Figure S22. ^1^H-NMR spectrum of compound **11**; Figure S23. ^13^C-NMR spectrum of compound **11**; Figure S24. HSQC spectrum of compound **11**; Figure S25. HMBC spectrum of compound **11**; Figure S26. ^1^H-NMR spectrum of compound **12**; Figure S27. ^1^H-NMR spectrum of compound **13**; Figure S28. ^13^C-NMR spectrum of compound **13**; Figure S29. ^1^H-NMR spectrum of compound **14**; Figure S30. ^13^C-NMR spectrum of compound **14**; Figure S31. ^1^H-NMR spectrum of compound **15**; Figure S32. ^13^C-NMR spectrum of compound **15**; Figure S33. ^1^H-NMR spectrum of compound **16**; Figure S34. ^13^C-NMR spectrum of compound **16**; Figure S35. HSQC spectrum of compound **16**; Figure S36. HMBC spectrum of compound **16**.

## References

[1] Dubéarnès A., Julius A., Utteridge T.M.A. (2015). A synopsis of the genus *Embelia* in peninsular Malaysia and Singapore. Studies in Malaysian Myrsinaceae III. Kew Bull..

[2] Vijayan K.R., Raghu A.V. (2021). Embelin: An HPTLC method for quantitative estimation in five species of genus *Embelia* Burm. f. Future J. Pharm. Sci..

[3] Sharma V., Gautam D.N.S., Radu A.F., Behl T., Bungau S.G., Vesa C.M. (2022). Reviewing the traditional/modern uses, phytochemistry, essential oils/extracts and pharmacology of *Embelia ribes* Burm. Antioxidants.

[4] Manguro L.O.A., Okwiri S.O., Lemmen P. (2006). Oleanane-type triterpenes of *Embelia schimperi* leaves. Phytochemistry.

[5] Bouzeko I.L.T., Ndontsa B.L., Nguekeu Y.M.M., Awouafack M.D., Wong C.P., Mpetga J.D.S., Mbouangouere R., Tane P., Morita H. (2019). A new alkylbenzoquinone from *Embelia rowlandii* Gilg. (Myrsinaceae). Nat. Prod. Res..

[6] Manguro L.O.A., Ugi I., Lemmen P. (2005). Flavonol glycosides from the leaves of *Embelia keniensis*. J. Chin. Chem. Soc..

[7] Qin Y., Chen J.P., Li C.Y., Zhu L.J., Zhang X., Wang J.H., Yao X.S. (2021). Flavonoid glycosides from the fruits of *Embelia ribes* and their anti-oxidant and *α*-glucosidase inhibitory activities. J. Asian Nat. Prod. Res..

[8] Dang P.H., Nguyen H.X., Nguyen N.T., Le H.N.T., Nguyen M.T.T. (2014). α-Glucosidase inhibitors from the stems of *Embelia ribes*. Phytother. Res..

[9] Dang P.H., Nguyen N.T., Nguyen H.X., Nguyen L.B., Le T.H., Van Do T.N., Can M.V., Nguyen M.T.T. (2015). α-Glucosidase inhibitors from the leaves of *Embelia ribes*. Fitoterapia.

[10] Yang L.J. (2016). Chemical constituents from the fruits of *Embelia laeta*. Chin. Pharm. J..

[11] Feng X., Li Y.H., Liang C.Y., Wang H., Zhang X.W. (2013). Chemical constituents from *Embelia laeta*. Zhong Yao Cai (J. Chin. Med. Mater.).

[12] Chen J.P., Zhu L.J., Su X.X., Zhang K.X., Zhang X., Wang J.H., Yao X.S. (2018). New alkylresorcinols from the fruits of *Embelia ribes*. Fitoterapia.

[13] Machocho A.K., Kiprono P.C., Grinberg S., Bittner S. (2003). Pentacyclic triterpenoids from *Embelia schimperi*. Phytochemistry.

[14] Manguro L.O.A., Ugi I., Lemen P. (2004). Further flavonol glycosides of *Embelia schimperi* leaves. Bull. Chem. Soc. Ethiop..

[15] Mahendran S., Badami S., Maithili V. (2011). Evaluation of antidiabetic effect of embelin from *Embelia ribes* in alloxan induced diabetes in rats. Biomed. Prev. Nutr..

[16] Bhandari U., Kanojia R., Pillai K.K. (2002). Effect of ethanolic extract of *Embelia ribes* on dyslipidemia in diabetic rats. J. Diabetes Res..

[17] Bhandari U., Jain N., Pillai K.K. (2007). Further studies on antioxidant potential and protection of pancreatic β-cells by *Embelia ribes* in experimental diabetes. J. Diabetes Res..

[18] Durg S., Veerapur V.P., Neelima S., Dhadde S.B. (2017). Antidiabetic activity of *Embelia ribes*, embelin and its derivatives: A systematic review and meta-analysis. Biomed. Pharmacother..

[19] Bhandari U., Chaudhari H.S., Khanna G., Najmi A.K. (2013). Antidiabetic effects of *Embelia ribes* extract in high fat diet and low dose streptozotocin-induced type 2 diabetic rats. Front. Life Sci..

[20] Bhandari U., Jain N., Ansari M.N., Pillai K.K. (2008). Beneficial effect of *Embelia ribes* ethanolic extract on blood pressure and glycosylated hemoglobin in streptozotocin-induced diabetes in rats. Fitoterapia.

[21] Chaudhari H.S., Bhandari U., Khanna G. (2012). Preventive effect of embelin from *Embelia ribes* on lipid metabolism and oxidative stress in high-fat diet-induced obesity in rats. Planta Med..

[22] Nazish I., Ansari S.H., Arora P. (2012). Antiobesity actions of *Embelia ribes*. Pharmacogn. J..

[23] Gupta G., Kazmi I., Afzal M., Upadhyay G., Singh R., Habtemariam S. (2013). Antidepressant-like activity of embelin isolated from *Embelia ribes*. Phytopharmacology.

[24] Thippeswamy B.S., Nagakannan P., Shivasharan B.D., Mahendran S., Veerapur V.P., Badami S. (2011). Protective effect of embelin from *Embelia ribes* Burm. against transient global ischemia-induced brain damage in rats. Neurotox. Res..

[25] Durg S., Kumar N., Vandal R., Dhadde S.B., Thippeswamy B.S., Veerapur V.P., Badami S. (2017). Antipsychotic activity of embelin isolated from *Embelia ribes*: A preliminary study. Biomed. Pharmacother..

[26] Dhayalan M., Denison M.I.J., L J.A., Krishnan K., N G.N. (2017). In vitro antioxidant, antimicrobial, cytotoxic potential of gold and silver nanoparticles prepared using *Embelia ribes*. Nat. Prod. Res..

[27] Afzal M., Gupta G., Kazmi I., Rahman M., Upadhyay G., Ahmad K., Imam F., Pravez M., Anwar F. (2012). Evaluation of anxiolytic activity of embelin isolated from *Embelia ribes*. Biomed. Aging Pathol..

[28] Ansari M.N., Bhandari U., Islam F., Tripathi C.D. (2008). Evaluation of antioxidant and neuroprotective effect of ethanolic extract of *Embelia ribes* Burm in focal cerebral ischemia/reperfusion-induced oxidative stress in rats. Fundam. Clin. Pharmacol..

[29] Swamy H.K., Krishna V., Shankarmurthy K., Rahiman B.A., Mankani K.L., Mahadevan K.M., Harish B.G., Naika H.R. (2007). Wound healing activity of embelin isolated from the ethanol extract of leaves of *Embelia ribes* Burm. J. Ethnopharmacol..

[30] Hossan M.S., Fatima A., Rahmatullah M., Khoo T.J., Nissapatorn V., Galochkina A.V., Slita A.V., Shtro A.A., Nikolaeva Y., Zarubaev V.V. (2018). Antiviral activity of *Embelia ribes* Burm. f. against influenza virus in vitro. Arch. Virol..

[31] Rambaran N., Naidoo Y., Dwarka D., Mellem J., Thimmegowda S.C., Baijnath H. (2024). In vitro antioxidant and anticancer potential of the vegetative and reproductive organs of *Embelia ruminata*. J. Herbs Spices Med. Plants.

[32] Lemmich J. (1996). Anthelmintic usage of extracts of *Embelia schimperi* from Tanzania. J. Ethnopharmacol..

[33] Li W., Liu X., Liang X.L., Chen Y., Pang D., Huang Z., Han Q., Liu W., Chen L. (2020). Optimization of extraction process and antioxidant activity of polysaccharide in *Embelia parviflora* Wall. by RSM. Med. Plant.

[34] Anh M.T.H., Mau C.H., Thuong S.D. (2025). Morphological characteristics and sequence of trnE-trnT of Embelia parviflora Wall. ex A. DC.). TNU J. Sci. Technol..

[35] Lu S.H., Li Y.H., Chen Y., Zeng H.S., Zhang L., Zheng X.R. (2012). Study on the chemical constituents of volatile oil from different parts of *Embelia parviflora* Wall. ex A. DC by GC-MS. Med. Plant.

[36] Wei J., Chen Y., Ma X., Qiu M., Qing B., Jiang L., Wen Z., Qin Z. (2024). Anti-inflammatory effect of polysaccharide from *Embelia parviflora* Wall, on rheumatoid arthritis in rats. Med. Plant.

[37] Liu W., Que Z., Li J., Chen Z., Huang Z., Pang D., Chen L., Chen Y. (2020). Study on sreening of effective components of *Embelia parviflora* for tonifying blood and its mechanism. China Pharm..

[38] Oanh V.T.K., Ha N.T.T., Duc H.V., Thuc D.N., Hang N.T.M., Thanh L.N. (2021). New triterpene and *nor*-diterpene derivatives from the leaves of *Adinandra poilanei*. Phytochem. Lett..

[39] Phuong D.T.L., Van Phuong N., Le Tuan N., Cong N.T., Hang N.T., Thanh L.N., Bankova V. (2023). Antimicrobial, cytotoxic, and *α*-glucosidase inhibitory activities of ethanol extract and chemical constituents isolated from *Homotrigona apicalis* propolis—In vitro and molecular docking studies. Life.

[40] Sosińska E., Przybylski R., Hazendonk P., Zhao Y.Y., Curtis J.M. (2013). Characterisation of non-polar dimers formed during thermo-oxidative degradation of *β*-sitosterol. Food Chem..

[41] Faizi S., Ali M., Saleem R., Irfanullah B.S. (2001). Complete ^1^H and ^13^C NMR assignments of stigma-5-en-3-*O*-β-glucoside and its acetyl derivative. Magn. Reson. Chem..

[42] Lavaud C., Massiot G., Moretti C., Le Men-Olivier L. (1994). Triterpene saponins from *Myrsine pellucida*. Phytochemistry.

[43] Acebey-Castellon I.L., Voutquenne-Nazabadioko L., Doan Thi Mai H., Roseau N., Bouthagane N., Muhammad D., Lavaud C. (2011). Triterpenoid saponins from Symplocos lancifolia. J. Nat. Prod..

[44] Li Y.L., Li J., Wang N.L., Yao X.S. (2008). Flavonoids and a new polyacetylene from *Bidens parviflora* Willd. Molecules.

[45] Ngoc L.T., Mai N.T. (2014). Chemical constituents of *Saraca dives*. Vietnam J. Sci. Technol..

[46] Thanh N.T.V., Hien D.T.T., Minh T.T., Cuong H.D., Nhiem N.X., Yen P.H., Van Kiem P. (2019). Quercetin glycosides and sesquiterpenes from *Phoebe poilanei* Kosterm. Vietnam J. Chem..

[47] Zhong X.N., Otsuka H., Ide T., Hirata E., Takushi A., Takeda Y. (1997). Three flavonol glycosides from leaves of *Myrsine seguinii*. Phytochemistry.

[48] Niu C., Yang L.P., Zhang Z.Z., Zhou D.J., Kong J.C., Liu Z.Q., Wang Z.H. (2022). Chemical constituents of *Ampelopsis japonica*. Chem. Nat. Comp..

[49] Wang H., Cong W.L., Fu Z.L., Chen D.F., Wang Q. (2017). Anti-complementary constituents of *Viola kunawarensis*. Nat. Prod. Res..

[50] Wu Q.L., Wang M., Simon J.E., Yu S.C., Xiao P.G., Ho C.T. (2003). Studies on the chemical constituents of loquat leaves (*Eriobotrya japonica*). Orient. Food Herbs.

[51] Ren Y., Anaya-Eugenio G.D., Czarnecki A.A., Ninh T.N., Yuan C., Chai H.B., Soejarto D.D., Burdette J.E., de Blanco E.J.C., Kinghorn A.D. (2018). Cytotoxic and NF-κB and mitochondrial transmembrane potential inhibitory pentacyclic triterpenoids from *Syzygium corticosum* and their semi-synthetic derivatives. Bioorg. Med. Chem..

[52] Huong P.T.T., Nguyen V.T., Diep C.N., Thao N.P., Cuong N.X., Nam N.H., Minh C.V. (2015). Antimicrobial compounds from *Rhizophora stylosa*. Vietnam J. Sci. Technol..

[53] Khallouki F., Hull W.E., Würtele G., Haubner R., Erben G., Owen R.W. (2019). Isolation of the major phenolic compounds in the pits of brined green olive drupes: Structure elucidation by comprehensive ^1^H/^13^C-NMR spectroscopy. Nat. Prod. Comm..

[54] Itoh A., Isoda K., Kondoh M., Kawase M., Watari A., Kobayashi M., Tamesada M., Yagi K. (2010). Hepatoprotective effect of syringic acid and vanillic acid on CCl4-induced liver injury. Biol. Pharm. Bull..

[55] Raffauf R.F., Zennie T.M., Onan K.D., Le Quesne P.W. (1984). Funebrine, a structurally novel pyrrole alkaloid, and other. gamma.-hydroxyisoleucine-related metabolites of *Quararibea funebris* (Llave) Vischer (Bombacaceae). J. Org. Chem..

[56] Trang B.T., Thuong C.H., Thao P.X., Mac D.H., Gree R. (2021). A new approach for the synthesis of sotolon in racemic and enantioenriched forms. Vietnam J. Chem..

[57] Ali Z., Khan I.A. (2011). Alkyl phenols and saponins from the roots of *Labisia pumila* (Kacip Fatimah). Phytochemistry.

[58] Row L.C., Chen C.M., Ho J.C. (2003). Two cerebrosides and one acylglycosyl sterol from *Monochoria vaginalis*. J. Chin. Chem. Soc..

[59] Zhang G.L., Li N., Wang Y.H., Zheng Y.T., Zhang Z., Wang M.W. (2007). Bioactive lignans from *Peperomia heyneana*. J. Nat. Prod..

[60] Jiang J., Sun C., Wang G., Xu Q., Bian Y., Li J., Li J., Ding R., Lin H., Tian W. (2025). C-13 Norisoprenoids and Eudesmanoids from *Nelumbo nucifera* Gaertn. Regulate the Lipid Metabolism via the AMPK/ACC/SREBP-1c Signaling Pathway. Chem. Biodiver..

[61] Liu J.J., Hao J.J., Tan M., Liao C.C., Liu D., Li H.M., Li R.T. (2024). Iridoids and other constituents from the leaves and stems of *Valeriana officinalis* var. latifolia. Phytochemistry.

[62] Wang L.J., Xiong J., Zou Y., Mei Q.B., Wang W.X., Hu J.F. (2016). Sesquiterpenoids from the Chinese endangered plant *Manglietia aromatica*. Phytochem. Lett..

[63] Tu P.C., Tseng H.C., Liang Y.C., Huang G.J., Lu T.L., Kuo T.F., Kuo Y.H. (2019). Phytochemical investigation of *Tradescantia albiflora* and anti-inflammatory butenolide derivatives. Molecules.

[64] Pan Y.B., Song X.Q., Sun P., Zhang H. (2023). Chemical constituents from the stems of *Marsdenia tenacissima*. Phytochem. Lett..

[65] Liu Q.W., Chen C.H., Wang X.F., Jiang K., Qu S.J., Dai Y.R., Tan C.H. (2019). Triterpenoids, megastigmanes and hydroxycinnamic acid derivatives from *Anisomeles indica*. Nat. Prod. Res..

[66] Yang H.H., Oh K.E., Jo Y.H., Ahn J.H., Liu Q., Turk A., Jang J.Y., Hwang B.Y., Lee M.K. (2018). Characterization of tyrosinase inhibitory constituents from the aerial parts of *Humulus japonicus* using LC-MS/MS coupled online assay. Bioorg. Med. Chem..

[67] DellaGreca M., Di Marino C., Zarrelli A., D’Abrosca B. (2004). Isolation and phytotoxicity of apocarotenoids from *Chenopodium album*. J. Nat. Prod..

[68] Wang H.L., Ding J. (2020). Extraction Method of Neoflavonoid Compound. Patent.

[69] Otsuka H., Yao M., Kamada K., Takeda Y. (1995). Alangionosides G-M: Glycosides of megastigmane derivatives from the leaves of *Alangium premnifolium*. Chem. Pharm. Bull..

[70] De Tommasi N., Piacente S., De Simone F., Pizza C. (1996). Constituents of *Cydonia vulgaris*: Isolation and structure elucidation of four new flavonol glycosides and nine new α-ionol-derived glycosides. J. Agric. Food Chem..

[71] Srinroch C., Sahakitpichan P., Techasakul S., Chimnoi N., Ruchirawat S., Kanchanapoom T. (2019). 2-Aminobenzoyl and megastigmane glycosides from *Wrightia antidysenterica*. Phytochem. Lett..

[72] Michalska K., Galanty A., Le T.N., Malarz J., Vuong N.Q., Pham V.C., Stojakowska A. (2021). New polyesterified ursane derivatives from leaves of *Maesa membranacea* and their cytotoxic activity. Molecules.

[73] Manome T., Hara Y., Ishibashi M. (2023). A new 1, 2-diketone physalin isolated from *Physalis minima* and TRAIL-resistance overcoming activity of physalins. J. Nat. Med..

[74] Ninh B.H., Dung D.T., Tai B.H., Yen P.H., Nhiem N.X., Hien T.T.T., Kiem P.V. (2023). New isopropyl chromone and flavanone glucoside compounds from the leaves of *Syzygium cerasiforme* (Blume) Merr. & LM Perry and their inhibition of nitric oxide production. Chem. Biodiver..

[75] Thao N.P., Linh K.T.P., Quan N.H., Trung V.T., Binh P.T., Cuong N.T., Nam N.H., Thanh N.V. (2022). Cytotoxic metabolites from the leaves of the mangrove *Rhizophora apiculata*. Phytochem. Lett..

[76] Kornpointner C., Hochenegger N.J., Shi B.B., Berger A., Theiner J., Brecker L., Schinnerl J. (2022). Phytochemistry meets geochemistry—Blumenol C sulfate: A new megastigmane sulfate from *Palicourea luxurians* (Rubiaceae: Palicoureeae). Molecules.

[77] Thapsut M., Singha S., Seeka C., Sutthivaiyakit S. (2021). Megastigmanes from the aerial part of *Euphorbia heterophylla*. Phytochem. Lett..

[78] Uyen N.H., Widyowati R., Sulistyowaty M.I., Sugimoto S., Yamano Y., Kawakami S., Otsuka H., Matsunami K. (2020). Firmosides A and B: Two new sucrose ferulates from the aerial parts of Silene firma and evaluation of radical scavenging activities. J. Nat. Med..

[79] Deng L., Zong W., Tao X., Liu S., Feng Z., Lin Y., Liao Z., Chen M. (2019). Evaluation of the therapeutic effect against benign prostatic hyperplasia and the active constituents from *Epilobium angustifolium* L.. J. Ethnopharmacol..

[80] Lin Q.M., Wang Y., Yu J.H., Liu Y.L., Wu X., He X.R., Zhou Z.W. (2019). Tyrosinase inhibitors from the leaves of *Eucalyptus globulus*. Fitoterapia.

[81] Yan J., Shi X., Donkor P.O., Zhu H., Gao X., Ding L., Qiu F. (2017). Nine pairs of megastigmane enantiomers from the leaves of *Eucommia ulmoides* Oliver. J. Nat. Med..

[82] Phetkul U., Hayiawae N., Khunthong S., Daus M., Voravuthikunchai S.P., Tamvapee P., Watanapokasin R., Chakthong S. (2023). Zanthoisobutylamides A−C: Rare dimeric C-6 substituent dihydrobenzophenanthridine alkaloids from the roots of *Zanthoxylum nitidum*. Nat. Prod. Res..

[83] Rahim A., Saito Y., Miyake K., Goto M., Nakagawa-Goto K. (2021). Novel seco-phenanthroquinolizidine alkaloids from Indonesian *Boehmeria virgata*. Phytochem. Lett..

[84] Shen D.Y., Kuo P.C., Huang S.C., Hwang T.L., Chan Y.Y., Shieh P.C., Ngan N.T., Thang T.D., Wu T.S. (2017). Constituents from the leaves of *Clausena lansium* and their anti-inflammatory activity. J. Nat. Med..

[85] Vitek R., de Novais L.M., Torquato H.F., Paredes-Gamero E.J., de Carvalho M.G., de Sousa P.T., Jacinto M.J., da Silva V.C. (2017). Chemical constituents and antileukemic activity of *Eugenia dysenterica*. Nat. Prod. Res..

[86] Lee M., Lee H.H., Lee J.K., Ye S.K., Kim S.H., Sung S.H. (2013). Anti-adipogenic activity of compounds isolated from *Idesia polycarpa* on 3T3-L1 cells. Bioorg. Med. Chem. Lett..

[87] Sob S.V.T., Wabo H.K., Tang C.P., Tane P., Ngadjui B.T., Ye Y. (2011). Phenol esters and other constituents from the stem barks of *Stereospermum acuminatissimum*. J. Asian Nat. Prod. Res..

[88] Tip-pyang S., Limpipatwattana Y., Khumkratok S., Siripong P., Sichaem J. (2010). A new cytotoxic 1-azaanthraquinone from the stems of *Goniothalamus laoticus*. Fitoterapia.

[89] Chiang Y.M., Liu H.K., Lo J.M., Chien S.C., Chan Y.F., Lee T.H., Su J.K., Kuo Y.H. (2003). Cytotoxic constituents of the leaves of *Calocedrus formosana*. J. Chin. Chem. Soc..

[90] Pan W.B., Chang F.R., Wei L.M., Wu Y.C. (2003). New flavans, spirostanol sapogenins, and a pregnane genin from *Tupistra chinensis* and their cytotoxicity. J. Nat. Prod..

[91] Chen C.Y., Chang F.R., Teng C.M., Wu Y.C. (1999). Cheritamine, A new N-fatty acyl tryptamine and other constituents from the stems of *Annona cherimola*. J. Chin. Chem. Soc..

[92] Van Nguyen Thien T., Do L.T.M., Dang P.H., Huynh N.V., Dang H.P., Nguyen T.T., Tran K.T., Huu N.D.M., That Q.T. (2021). A new lignan from the flowers of Hibiscus sabdariffa L. (Malvaceae). Nat. Prod. Res..

[93] Sheng Z., Dai H., Pan S., Wang H., Hu Y., Ma W. (2014). Isolation and characterization of an *α*-glucosidase inhibitor from Musa spp.(Baxijiao) flowers. Molecules.

[94] Ding H., Hu X., Xu X., Zhang G., Gong D. (2018). Inhibitory mechanism of two allosteric inhibitors, oleanolic acid and ursolic acid on *α*-glucosidase. Inter. J. Biol. Macromol..

[95] Peng X., Zhang G., Liao Y., Gong D. (2016). Inhibitory kinetics and mechanism of kaempferol on *α*-glucosidase. Food Chem..

[96] Van Do T.N., Nguyen H.X., Truong T.Q., Ly D.T., Dang N.M.T., Le T.H., Nguyen M.T.T. (2024). Some terpenoid compounds from the ethyl acetate extract of *Annona muricata* L. leaves and their *α*-glucosidase inhibitory activity. Sci. Technol. Dev. J. Nat. Sci..

[97] Tabussum A., Riaz N., Saleem M., Ashraf M., Ahmad M., Alam U., Jabeen B., Malik A., Jabbar A. (2013). α-Glucosidase inhibitory constituents from Chrozophora plicata. Phytochem. Lett..

[98] Liu Y., Zhan L., Xu C., Jiang H., Zhu C., Sun L., Sun C., Li X. (2020). α-Glucosidase inhibitors from Chinese bayberry (*Morella rubra* Sieb. et Zucc.) fruit: Molecular docking and interaction mechanism of flavonols with different B-ring hydroxylations. RSC Adv..

[99] Wang J., Zhao J., Yan Y., Liu D., Wang C., Wang H. (2020). Inhibition of glycosidase by ursolic acid: In vitro, in vivo and in silico study. J. Sci. Food Agric..

[100] Khusnutdinova E.F., Petrova A.V., Thu H.N.T., Tu A.L.T., Thanh T.N., Thi C.B., Bavkov D., Kazakova O.B. (2019). Structural modifications of 2,3-indolobetulinic acid: Design and synthesis of highly potent *α*-glucosidase inhibitors. Bioorg. Chem..

[101] Li N., Yang J., Wang C., Wu L., Liu Y. (2023). Screening bifunctional flavonoids of anti-cholinesterase and anti-glucosidase by in vitro and in silico studies: Quercetin, kaempferol and myricetin. Food Biosci..

