# Supplementary material for: Chemical Constituents and α-Glucosidase Inhibitory Activities of the Leaves of Embelia parviflora—In Vitro and In Silico Studies
Source: Life (Basel). 2025 Apr 22;15(5):680. doi: 10.3390/life15050680 (PMC12112773; doi:10.3390/life15050680)
Supplement: Supplementary file 1 [file life-15-00680-s001.zip › life-3551085-supplementary.pdf]

SUPPLEMENTARY MATERIAL

Chemical Constituents and *α*-Glucosidase Inhibitory Activities of Leaves of *Embelia parviflora* - *In vitro* and *In silico* Studies

Sy Danh Thuong[1,*], Mai Thi Hoang Anh[1], Nguyen Van Phuong[2], Chu Hoang Mau[1], Nguyen Huu Quan[1], Nguyen Thanh Cong[3], Le Nguyen Thanh[4,*]

[1] Thainguyen University of Education, Thai Nguyen University, Thai Nguyen 250000, Vietnam

[2] Department of Pharmacognosy, Faculty of Pharmacognosy and Traditional Medicine, Hanoi University of Pharmacy, Hanoi, 10000, Vietnam

[3] Department of Pharmacy, Dai Nam University, Hanoi, Vietnam

[4] National Institute of Medicinal Materials, Ministry of Health, Hanoi 10000, Vietnam

* Correspondence: thuongsd@tnue.edu.vn (Sy Danh Thuong)

lethanh@imbc.vast.vn (*Le Nguyen Thanh)*

Physical and Spectroscopic Data of Compounds

***β*-Sistosterol (1)**: White crystal; ESI-MS *m/z* 415 $[M+H]^+$. ^{1}H-NMR (500MHz, $CDCl_3$), δ (ppm): 5.34 (1H, br d, *J* = 4.5 Hz), 3.52 (1H, m), 1.01 (3H, s), 0.92 (3H, d, *J* = 6.5 Hz), 0.84 (3H, t, *J* = 7.5Hz), 0.83 (3H, d, J = 7.0 Hz), 0.80 (3H, d, *J* = 7.0 Hz), 0.68 (3H, s).

Daucosterol (2): White solid. ESI-MS *m/z* 577 $[M+H]^+$. ^{1}H-NMR (500 MHz, $CDCl_3$ +CD_3OD) *δ* (ppm): 5.35 (1H, br s, H-6), 4.41 (1H, d, *J* = 8.0 Hz, H-1′), 3.84 (1H, dd, *J* = 12.0 Hz, 3.0 Hz, H-6′a), 3.77 (1H, dd, *J* = 12.0 Hz, 4.5 Hz, H-6′b), 3.60-3.23 (5H, m, H-2′, H-3′, H-4′, H-5′ and H-3), 1.00 (3H, s, H-19), 0.94 (3H, d, *J* = 6.5 Hz, H-21), 0.86 (3H, t, *J* = 7.0 Hz, H-29), 0.85 (3H, d, *J* = 7.0 Hz, H-27), 0.83 (3H, d, *J* = 7.0 Hz, H-26), 0.68 (3H, s, H-18). ^{13}C-NMR (125 MHz, $CDCl_3$+CD_3OD) *δ* (ppm): 37.3 (C-1), 28.3 (C-2), 79.3 (C-3), 38.8 (C-4), 140.4 (C-5), 122.3 (C-6), 31.9 (C-7), 32.0 (C-8), 50.3 (C-9), 36.8 (C-10), 21.2 (C-11), 39.9 (C-12), 42.4 (C-13), 56.2 (C-14), 23.2 (C-15), 24.4 (C-16), 56.8 (C-17), 12.0 (C-18), 19.4 (C-19), 36.2 (C-20), 18.8 (C-21), 33.3 (C-22), 23.2 (C-23), 45.9 (C-24), 26.2 (C-25), 19.1 (C-26), 19.9 (C-27), 23.2 (C-28), 11.9 (C-29), 101.2 (C-1′), 73.6 (C-2′), 76.4 (C-3′), 75.8 (C-5′), 70.1 (C-4′), 61.9 (C-6′)

.

3-*O*-(6′-*O*-Palmitoyl)-*β*-D-glucopyranosyl stigmasterol (**3**): Colorless oil. ESI-MS *m/z* 813 $[M+H]^+$. ^{1}H-NMR (600 MHz, $CDCl_3$) *δ* (ppm): 5.17 (1H, dd, *J* = 15.0 Hz, 8.4 Hz, H-22), 5.16 (1H, br s, H-6), 5.03 (1H, dd, *J* = 15.0 Hz, 9.0 Hz, H-23), 4.47 (1H, dd, *J* = 12.0 Hz, 4.8 Hz, H-6′a), 4.38 (1H, d, *J* = 7.8 Hz, H-1′), 4.28 (1H, dd, *J* = 12.0 Hz, 1.8 Hz, H-6′b), 3.62 (1H, m, H-3), 3.56-3.33 (4H, m, H-2′, H-3′, H-4′, H-5′), 2.35 (2H, t, *J* = 7.8 Hz, H-2″), 1.02 (3H, d, *J* = 7.8 Hz, H-21), 0.88 (3H, t, *J* = 7.2 Hz, H-16″), 0.84 (3H, d, *J* = 6.6 Hz, H-27), 0.81 (3H, t, *J* = 7.2 Hz, H-29), 0.80 (3H, s, H-19), 0.80 (3H, d, *J* = 6.6 Hz, H-26), 0.55 (3H, s, H-18). ^{13}C-NMR (125 MHz, $CDCl_3$) *δ* (ppm): 37.1 (C-1), 29.5 (C-2), 78.9 (C-3), 34.4 (C-4), 139.5 (C-5), 117.3 (C-6), 31.8 (C-7), 31.9 (C-8), 49.4 (C-9), 34.2 (C-10), 21.5 (C-11), 39.4 (C-12), 43.2 (C-13), 55.1 (C-14), 23.0 (C-15), 28.5 (C-16), 55.9 (C-17), 12.0 (C-18), 19.0 (C-19), 40.8 (C-20), 21.4 (C-21), 138.1 (C-22), 129.5 (C-23), 51.3 (C-24), 40.2 (C-25), 21.0 (C-26), 12.9 (C-27), 22.6 (C-28), 12.2 (C-29), 101.1 (C-1′), 73.9 (C-2′), 73.6 (C-3′), 70.1 (C-4′), 75.9 (C-5′), 63.1 (C-6′), 174.6 (C-1″), 34.3 (C-2″), 24.9 (C-3″), 29.7-29.3 (C-4″-C-15″), 14.1 (C-16″).

Ursolic acid (4): White solid, ESI-MS: *m/z* 457 $[M+H]^+$, CTPT $C_{30}H_{48}O_3$ (M = 456). ^{1}H-NMR (500 MHz, $CDCl_3$+ CD_3OD) *δ* (ppm): 5.24 (1H, t, *J* = 7.5 Hz, H-12), 3.18 (1H, m, H-3), 1.08 (3H, s, H-27), 0.98 (3H, s, H-23), 0.93 (3H, s, H-24),0.92 (3H, d, *J* = 6.5 Hz, H-29), 0.86 (3H, d, *J* = 6.5

Hz, H-30), 0.81 (3H, s, H-26), 0.77 (3H, s, H-25),. ^{13}C-NMR (125 MHz, $CDCl_3$+ CD_3OD): 180.7 (C-28), 138.0 (C-13), 125.4 (C-12), 78.7 (C-3), 55.1 (C-5), 52.6 (C-18), 47.7 (C-17), 47.4 (C-9), 41.9 (C-14), 39.3 (C-8), 38.9 (C-4), 38.7 (C-19), 38.57 (C-20), 38.53 (C-1), 36.8 (C-10), 36.6 (C-22), 32.9 (C-7), 30.5 (C-21), 27.9 (C-23), 27.9 (C-2), 26.7 (C-15), 24.0 (C-16), 23.3 (C-27), 23.1 (C-11), 21.0 (C-30), 18.1 (C-6), 16.85 (C-29), 16.76 (C-26), 15.4 (C-25), 15.2 (C-24).

Kaempferol (**5**): Yellow solid. ESI-MS *m/z* 287 $[M+H]^+$. ^{1}H NMR (500 MHz, CD_3OD) δ (ppm): 8.09 (2H, d, J = 8.5 Hz, H-2′, 6′), 6.92 (2H, d, J = 8.5 Hz, H-3′, 5′), 6.40 (1H, d, J = 2.0 Hz, H-8), 6.20 (1H, d, J = 2.0 Hz, H-6). ^{13}C NMR (125 MHz, CD_3OD) δ (ppm): 148.0 (C-2), 137.1 (C-3), 177.3 (C-4), 162.4 (C-5), 99.3 (C-6), 165.5 (C-7), 94.4 (C-8), 158.2 (C-9), 104.5 (C-10), 123.7 (C-1′), 130.6 (C-2′,6′), 160.5 (C-4′), 116.3 (C-3′,5′).

Kaempferin (6) Yellow solid. ^{1}H-NMR (500 MHz, CD_3OD): δ_H (ppm) 7.79 (2H, d, J = 8.5 Hz, H-2´, H-6´), 6.96 (2H, d, J = 8.5 Hz, H-3´, H-5´), 6.40 (1H, d, J = 2.0 Hz, H-8), 6.23 (1H, d, J = 2.0 Hz, H-6), 5.39 (1H, d, J = 2.0 Hz, H-1″), 4.24 (1H, m, H-2″), 3.73 (1H, m, H-3″), 3.36-3.33 (2H, m, H-4″, H-5″), 0.95 (1H, d, J = 5.5 Hz, H-6″). ^{13}C-NMR (125 MHz, CD_3OD): δ_C (ppm) 158.5 (C-2), 136.2 (C-3), 179.7 (C-4), 161.6 (C-5), 99.8 (C-6), 165.9 (C-7), 94.8 (C-8), 159.3 (C-9), 105.9 (C-10), 122.6 (C-1′), 131.9 (C-2′, C-6′), 116.5 (C-3′, C-5′), 163.1 (C-4′), 103.5 (C-1″), 73.2 (C-2″), 72.1 (C-3″), 72.0 (C-4″), 71.9 (C-5″), 17.6 (C-6″).

Quercitrin (7) Yellow solid. ESI MS *m/z* 465 $[M+H]^+$. ^{1}H NMR (500 MHz, CD_3OD) δ (ppm): 7.36 (1H, d, J = 2.0 Hz, H-2'), 7.33 (1H, dd, J = 2.0, 7.0 Hz, H-6'), 6.93 (1H, d, J = 7.0 Hz, H-5'), 6.39 (1H, d, J = 1.5 Hz, H-8), 6.22 (1H, d, J = 1.5 Hz, H-6), 5.37 (1H, d, J = 1.5 Hz, H-1"), 4.23 (1H, dd, J = 1.5, 2.5 Hz, H-2"), 3.76 (1H, dd, J = 3.0, 8.0 Hz, H-3"), 3.44 (1H, m, H-4"), 3.33 (1H, m, H-5"), 0.95 (3H, d, J = 5.0 Hz, H-6"). ^{13}C NMR (125 MHz, CD_3OD) δ (ppm): δ (ppm): 179.7 (C-4), 165.9 (C-7), 163.2 (C-5), 159.3 (C-2), 158.5 (C-9), 149.8 (C-4'), 146.4 (C-3'), 136.2 (C-3), 123.0 (C-1'), 122.9 (C-6'), 116.9 (C-5'), 116.4 (C-2'), 105.9 (C-10), 103.5 (C-1"), 99.8 (C-6), 94.7 (C-8), 73.3 (C-4"), 72.2 (C-3"), 72.0 (C-2"), 71.9 (C-5"), 17.6 (C-6").

Quercetin-3-rhamnoside-3′-glucoside (8) Yellow solid. ^{1}H NMR (600 MHz, CD_3OD) δ (ppm): 6.25 (1H, s, H-6), 6.45 (1H, s, H-8), 7.72 (1H, d, J = 1.8 Hz, H-2'), 7.06 (1H, d, J = 8.4 Hz, H-5'), 7.53 (1H, dd, J = 1.8, 8.4 Hz, H-6'), 5.39 (1H, d, J = 1.2 Hz, H-1"), 3.54 (1H, m, H-2"), 4.26 (1H, m, H-3"), 3.36 (1H, m, H-4"), 3.19 (1H, m, H-5"), 0.91 (3H, d, J = 6.0 Hz, H-6"), 4.90 (1H, d, J =7,2 Hz, H-1‴), 3.61 (1H, m, H-2‴), 3.59 (1H, m, H-3‴), 3.80 (1H, dd, 9,0; 3,0, H-4‴), 3.54 (1H, m,

H-5‴), 3.84 (1H, dd, 12,0; 2,4, H-6‴a), 3,96 (1H, d, 12.0, H-6‴b). ^{13}C NMR (150 MHz, CD_3OD) *δ* (ppm): δ (ppm): 159.0 (C-2), 135.7 (C-3), 179.2 (C-4), 162.5 (C-5), 100.5 (C-6), 166.3 (C-7), 95.3 (C-8), 158.3 (C-9), 105.6 (C-10), 122.8 (C-1'), 119.4 (C-2'), 146.6 (C-3'), 151.4 (C-4'), 117.6 (C-5'), 126.6 (C-6'), 103.0 (C-1''),70.7 (C-2''),71.6 (C-3''),72.8 (C-4''),72.1 (C-5''), 19.5 (C-6''), 104.2 (C-1'''), 74.5 (C-2'''), 77.1 (C-3'''), 71.8 (C-4'''), 77.9 (C-5'''), 61.9 (C-6''').

9-Hydroxy-4,7-megastigmadien-3-one (**9**): White solid, ^{1}H-NMR (600 MHz, $CDCl_3$) *δ* (ppm): 5.90 (1H, s, H-4), 5.67 (1H, dd, *J* = 6.0; 15.6 Hz, H-8), 5.67 (1H, dd, *J* = 9.0; 15.6 Hz, H-7), 4.35 (1H, quin, *J* = 6.0 Hz, 5.4 Hz, H-9), 2.52 (1H, d, *J* = 9.0 Hz, H-6), 2.33 (1H, d, *J* = 16.8 Hz, H-2a), 2.09 (1H, d, *J* = 16.8 Hz, H-2b), 1.89 (3H, s, H-13), 1.26 (3H, d, *J* = 6.0 Hz, H-10), 1.04 (3H, s, H-11), 0.98 (3H, s, H-12). ^{13}C-NMR (150 MHz, $CDCl_3$) *δ* (ppm): 36.2 (C-1), 47.5 (C-2), 199.1 (C-3), 125.9 (C-4), 161.6 (C-5), 55.4 (C-6), 126.7 (C-7), 138.5 (C-8), 68.4 (C-9), 23.6 (C-10), 27.1 (C-11), 27.9 (C-12), 23.5 (C-13).

Compound (**10**): White solid, ^{1}H-NMR (500 MHz, $CDCl_3$) *δ* (ppm): 2.00 (1H, m, H-2a), 1.38 (1H, m, H-2b), 4.34 (1H, m, H-3), 2.29 (1H, m, H-4a), 1.48 (1H, m, H-4b), 5.85 (1H, m, H-8), 2.18 (3H, s, H-10), 1.16 (3H, s, H-11), 1.38 (3H, s, H-12), 1.43 (3H, s, H-13). ^{13}C-NMR (125 MHz, $CDCl_3$) *δ* (ppm): 36.1 (C-1), 49.0 (C-2), 63.9 (C-3), 48.7 (C-4), 72.4 (C-5), 118.7 (C-6), 198.1 (C-7), 100.8 (C-8), 209.5 (C-9), 26.4 (C-10), 29.1 (C-11), 31.7 (C-12), 31.0 (C-13).

(6*R*,7*E*,9*R*)-9-Hydroxy-4,7-megastigmadien-3-on 9-*O*-*β*-D-apiofuranosyl(1->6)-*β*-D-glucopyranoside (11) White solid, ^{1}H-NMR (500 MHz, $CDCl_3$) *δ* (ppm): 2.46 (1H, m, H-2a), 2.10 (1H, m, H-2b), 5.94 (1H, s, H-4), 2.43 (1H, d, J = 6.6 Hz, H-6), 5.77 (1H, dd, *J* = 6.6; 16.6 Hz, H-7), 5.66 (1H, dd, *J* = 2.4; 16.6 Hz, H-8), 4.38 (1H, d, H-9), 1.31 (3H, d, J= 6.0 Hz, H-10), 1.02 (3H, s, H-11), 1.05 (3H, s, H-12), 0.97 (3H, s, H-13), 4.40 (1H, d, *J* = 7.8 Hz, H-1'), 3.40 (1H, d, *J* = 7.8 Hz, H-2'), 3.22 (1H, m, H-3'), 3.39 (1H, m, H-4'), 3.95 (1H, m, H-5'), 3.95 (1H, m, H-6'a), 3.64 (1H, m, H-6'b), 5.04 (1H, d, *J* = 2,4 Hz, H-1''), 3.40 (1H, m, H-2''), 4.01 (1H, d, *J* = 9.6 Hz, H-4''), 3.82 4.01 (1H, d, *J* = 9.6 Hz, H-4''), 3.61 (2H, s, H-5''). ^{13}C-NMR (125 MHz, $CDCl_3$) *δ* (ppm): 37.0 (C-1), 48.1 (C-2), 203.4 (C-3), 125.8 (C-4), 167.3 (C-5), 56.5 (C-6), 137.6 (C-7), 129.2 (C-8), 77.6 (C-9), 23.9 (C-10), 27.4 (C-11), 28.0 (C-12), 21.1 (C-13), 102.3 (C-1'), 74.9 (C-2'), 77.8 (C-3'), 77.1 (C-4'), 77.8 (C-5'), 68.5 (C-6'), 110.6 (C-1''),76.5 (C-2''), 80.4 (C-3''),74.7 (C-4''), 65.1 (C-5'').

Vomifoliol (12): Pale yellow solid, $[\alpha]^{25}_{D}$ +185 (*c* 0,12, MeOH) ($[\alpha]^{25}_{D}$ +142.0 (*c* 1,0, MeOH) [16]). ESI-MS *m/z*: 225 $[M+H]^+$. ^{1}H-NMR (500 MHz, CD_3OD) *δ* (ppm): 5.89 (1H, t, H-5), 5.82

(1H, m, H-8), 5.80 (1H, m, H-7), 4.34 (1H, dq, *J* = 7.8 Hz, 5,4 Hz, H-9), 2.53 (1H, d, *J* = 19,8 Hz, H-3a), 2.18 (1H, d, *J* = 19,8 Hz, H-3b), 1.94 (3H, s, H-13), 1.26 (3H, d, *J* = 7,8 Hz, H-10), 1.06 (3H, s, H-11), 1.03 (3H, s, H-12). ^{13}C-NMR (125 MHz, CD_3OD) δ (ppm): 42.4 (C-1), 50.7 (C-2), 201.2 (C-3), 127.1 (C-4), 167.4 (C-5), 79.9 (C-6), 130.1 (C-7), 136.9 (C-8), 68.7 (C-9), 23.8 (C-10), 24.5 (C-11), 23.4 (C-12), 19.5 (C-13).

Methyl *p*-coumarate (**13**): Pale yellow oil. ^{1}H NMR (500 MHz, acetone-d_6) δ (ppm): 7.59 (1H, d, *J* = 16.2 Hz, H-7), 7.52 (2H, d, *J* = 8.4 Hz, H-2, H-6), 6.88 (2H, d, *J* = 8.4 Hz, H-3, H-5), 6.32 (1H, d, *J* = 16.2 Hz, H-8), 3.71 (3H, s, OMe). ^{13}C NMR (125 MHz, acetone-d_6) δ (ppm): 126.6 (C-1), 130.8 (C-2, C-6), 116.7 (C-3, C-5), 160.8 (C-4), 145.1 (C-7), 115.0 (C-8), 168.0 (C-9), 51.5 (OCH_3).

Vanillic acid (**14**): Pale yellow solid. ^{1}H NMR (500 MHz, CD_3OD) δ (ppm): 7.57 (1H, d, *J* = 2.0 Hz, H-2), 7.56 (1H, dd, *J* = 2.0 Hz, 8.0 Hz, H-6), 6.85 (1H, d, *J* = 8.0 Hz, H-5), 3.91 (3H, s, OMe). ^{13}C NMR (125 MHz, CD_3OD) δ (ppm): 125.3 (C-1), 115,8 (C-2), 152.6 (C-3), 148.6 (C-4), 113.8 (C-5), 123,1 (C-6), 170.1 (COOH), 56.4 (OCH_3).

Syringic acid (15) Pale yellow solid. ^{1}H-NMR (500 MHz, CD_3OD) *δ:* 7.35 (2H, s, H-2,6), 3.90 (6H, s, OMe). ^{13}C-NMR (125 MHz, CD_3OD) *δ:* 123.4 (C-1), 108.3 (C-2, C-6), 148.7 (C-3, C-5), 141.2 (C-4), 170.9 (COOH).

Sotolone (**16**): Colorless oil. $[M+H]^+$. ^{1}H NMR (500 MHz, $CDCl_3$) δ (ppm): 4.81 (1H, q, *J* = 6,0 Hz, H-5), 4.04 (1H, br, OH), 1.88 (3H, s, Me-4), 1.40 (3H, d, *J* = 6,0 Hz, Me-5). ^{13}C NMR (125 MHz, $CDCl_3$) δ (ppm): 170.8 (C-2), 137.6 (C-3), 132.1 (C-4), 78.2 (C-5), 18.5 (Me-4), 9.1 (Me-5).

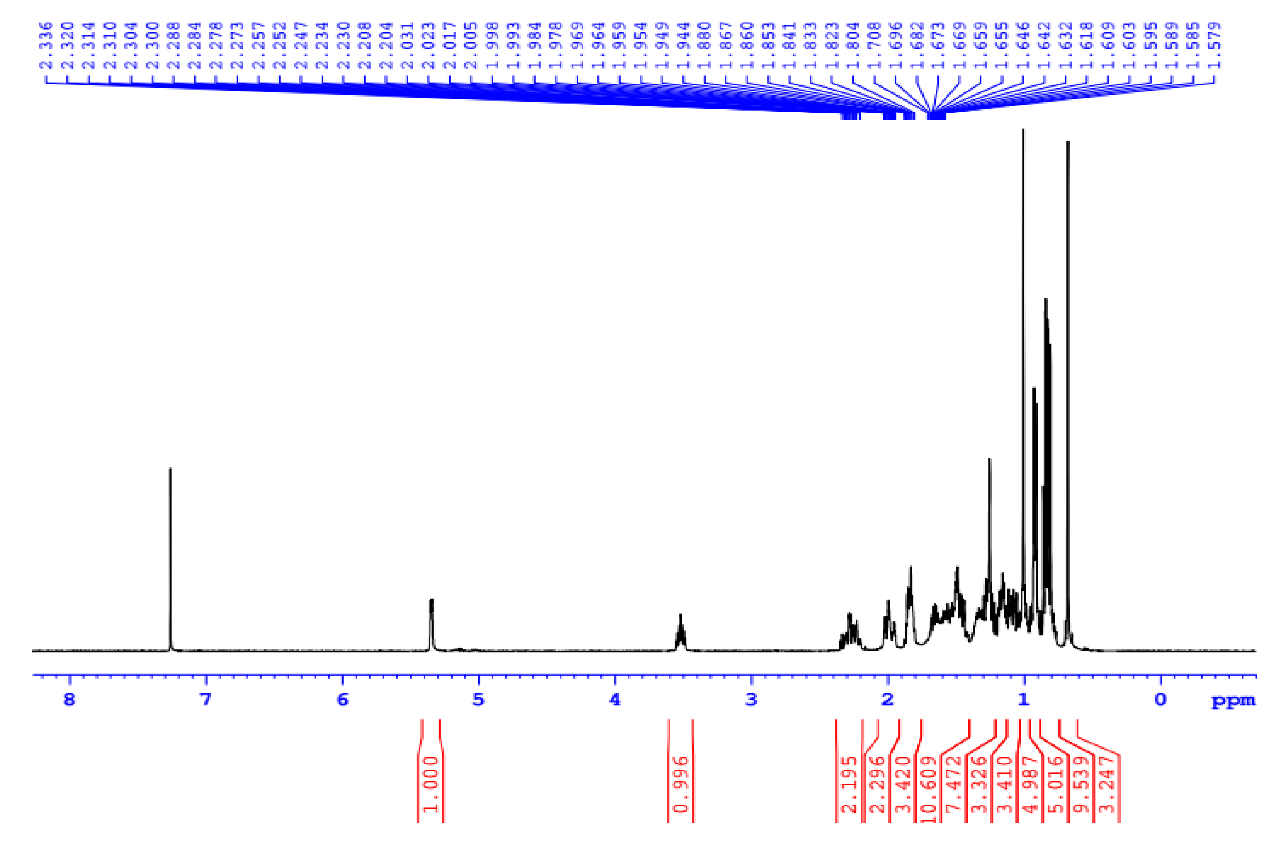

Figure S1. ^{1}H-NMR spectrum of compound **1**

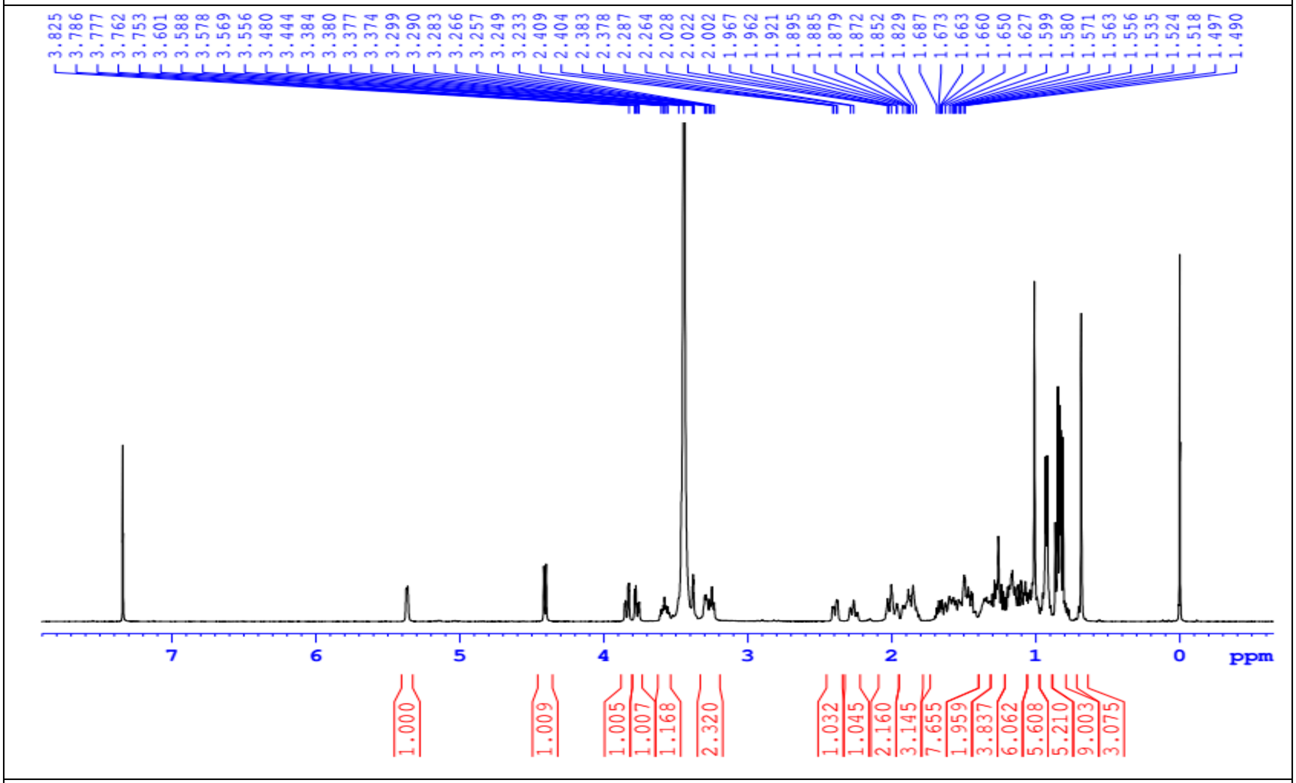

Figure S2. ^{1}H-NMR spectrum of compound **2**

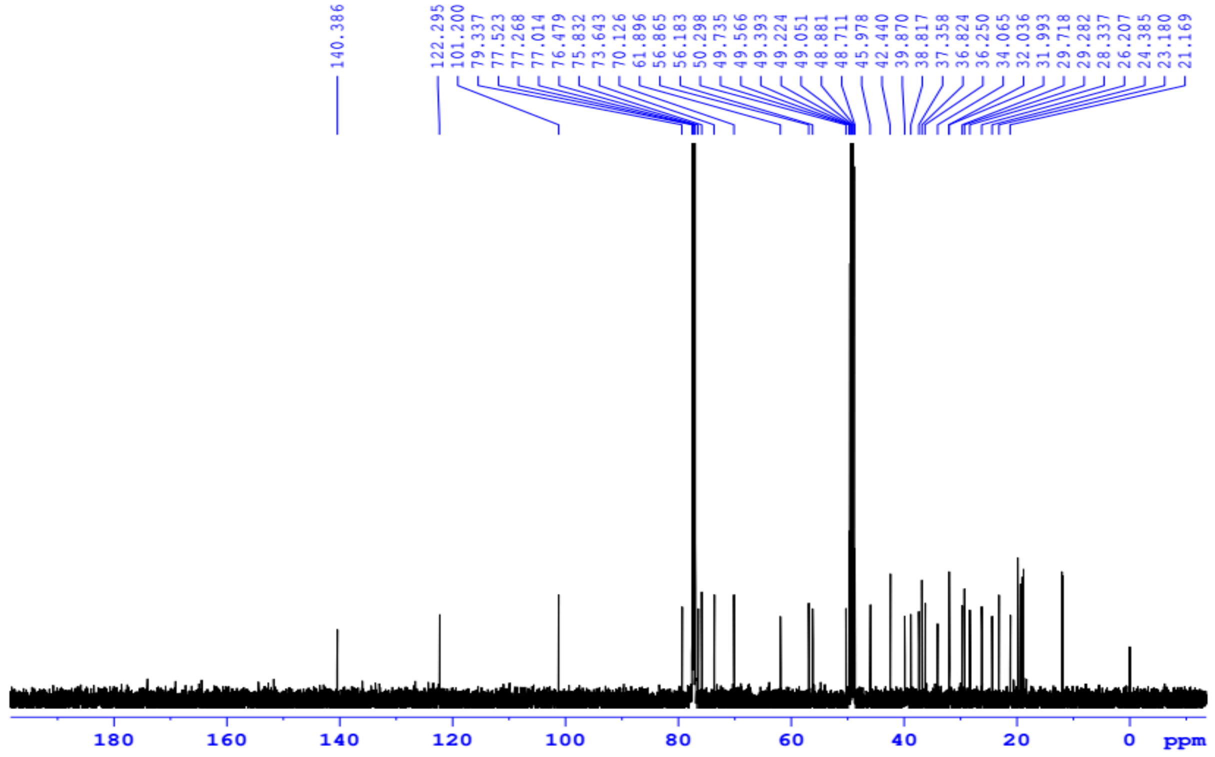

Figure S3. ^{13}C-NMR spectrum of compound **2**

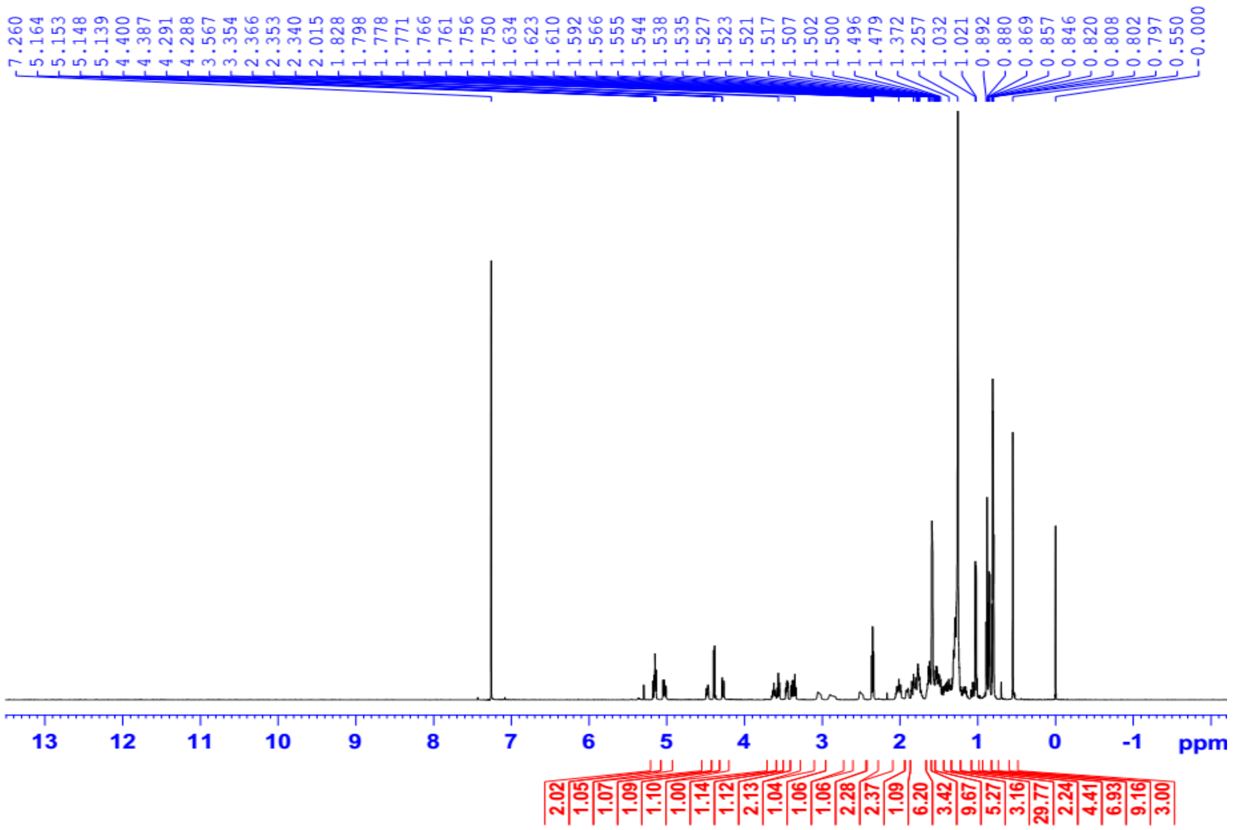

Figure S4. ^{1}H-NMR spectrum of compound **3**

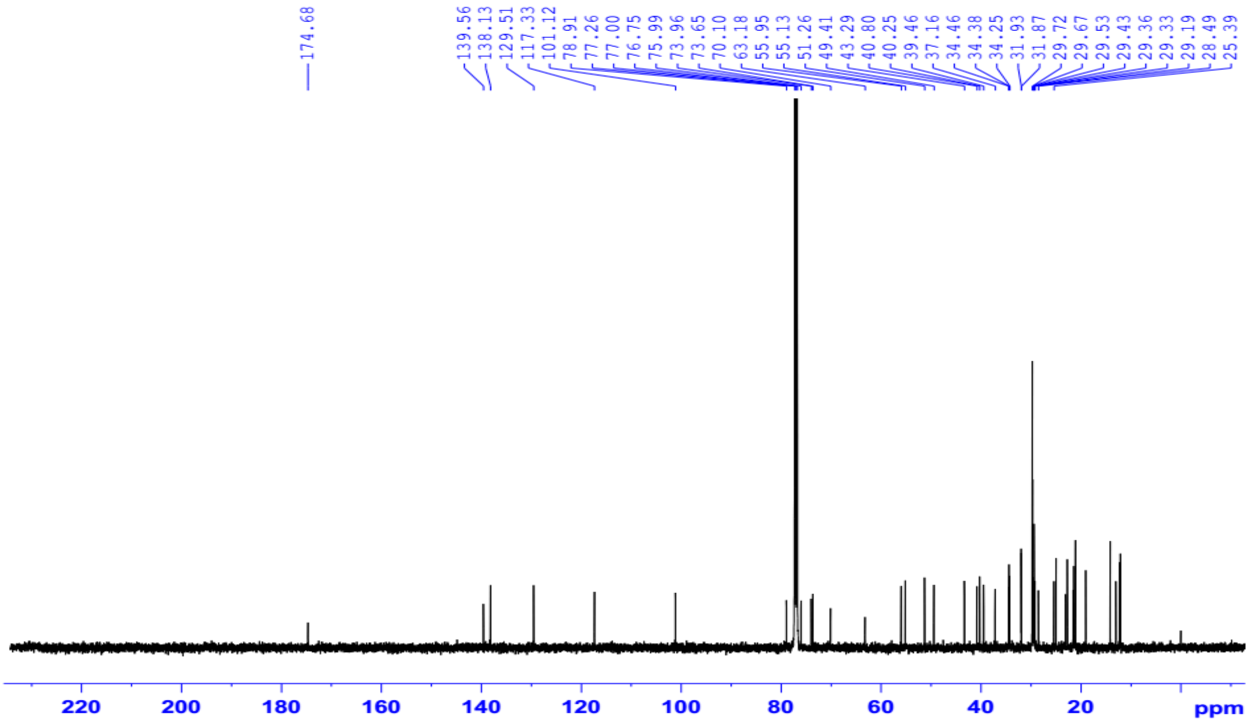

Figure S5. ^{13}C-NMR spectrum of compound **3**

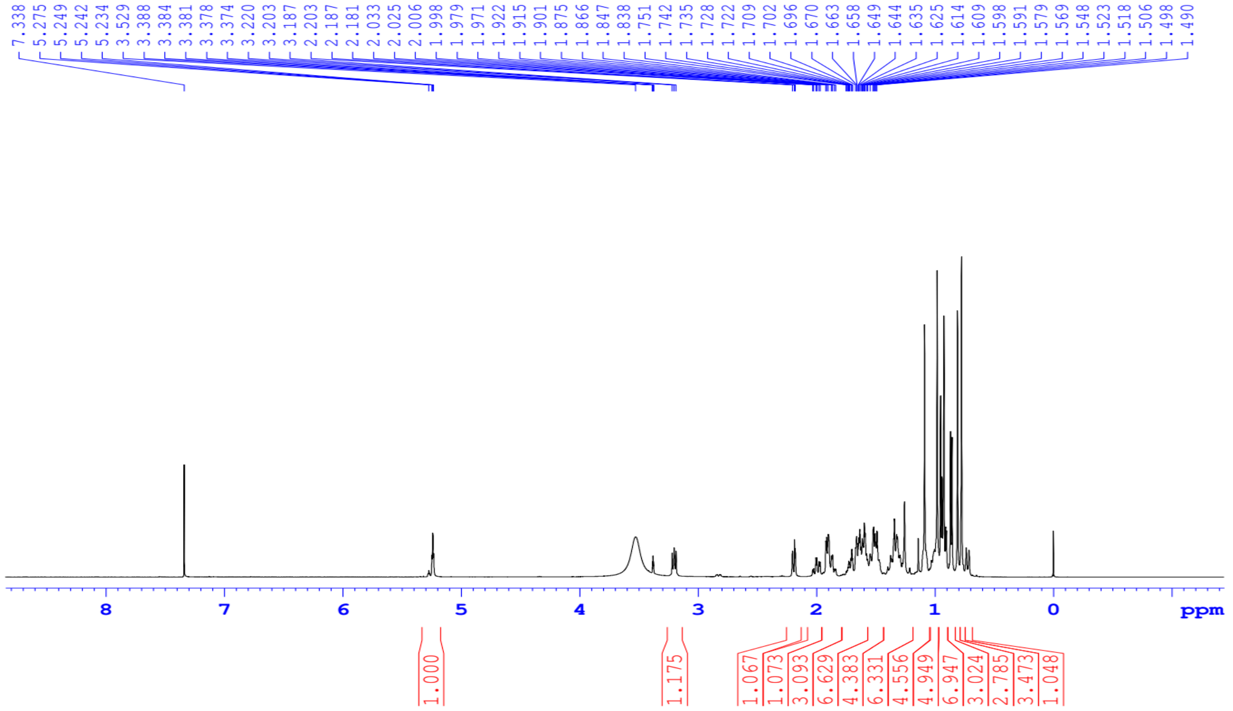

Figure S6. ^{1}H-NMR spectrum of compound **4**

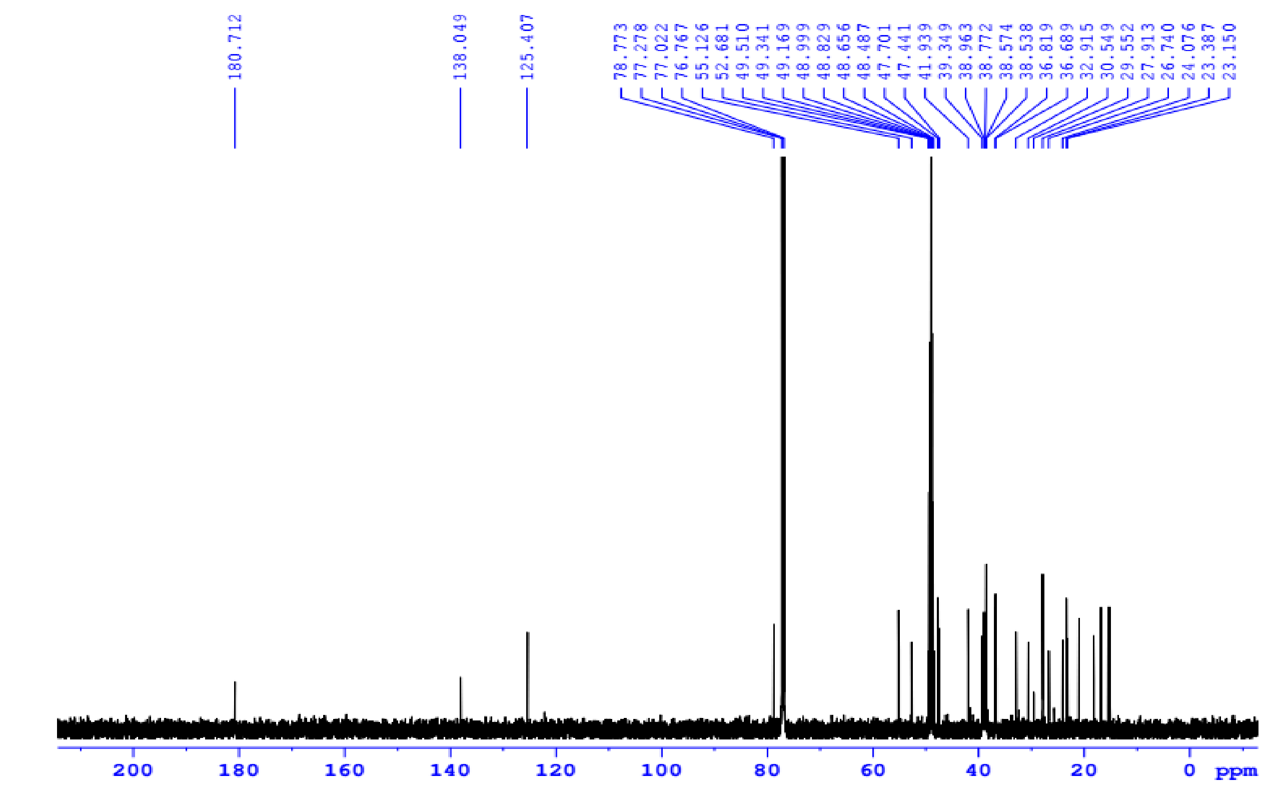

Figure S7. ^{13}C-NMR spectrum of compound **4**

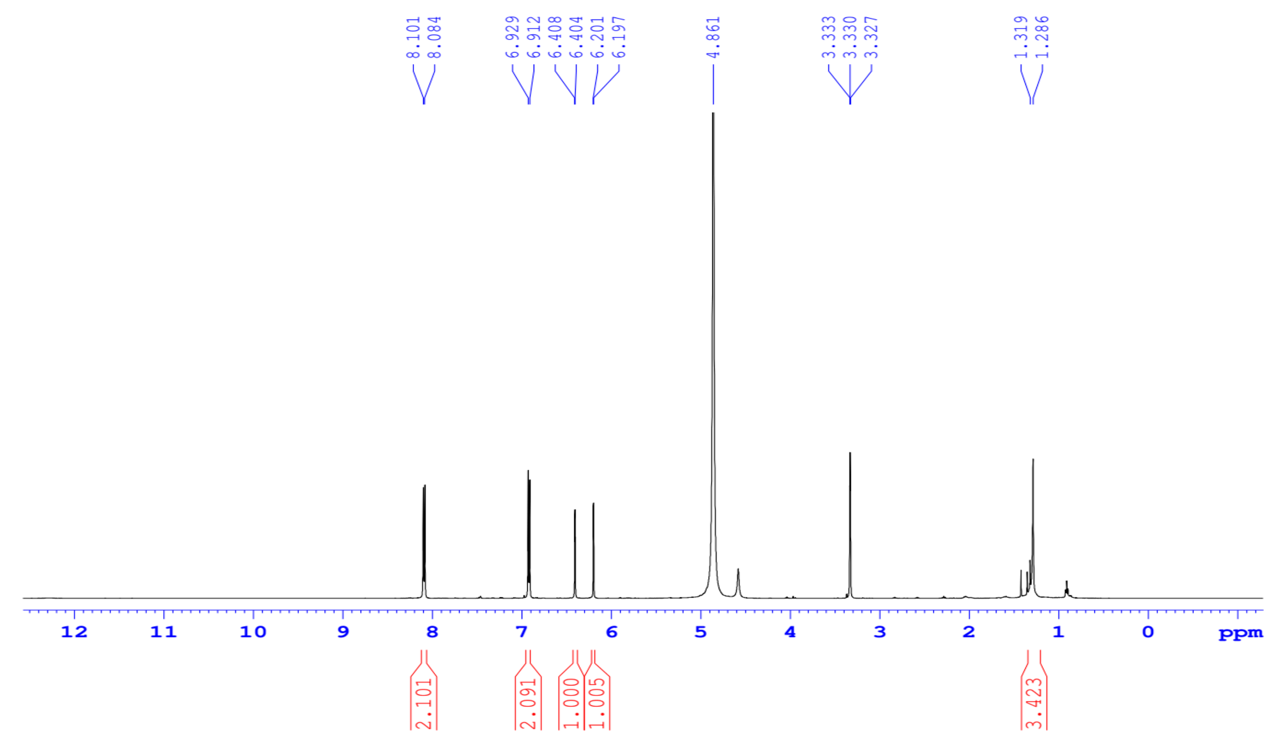

Figure S8. ^{1}H-NMR spectrum of compound **5**

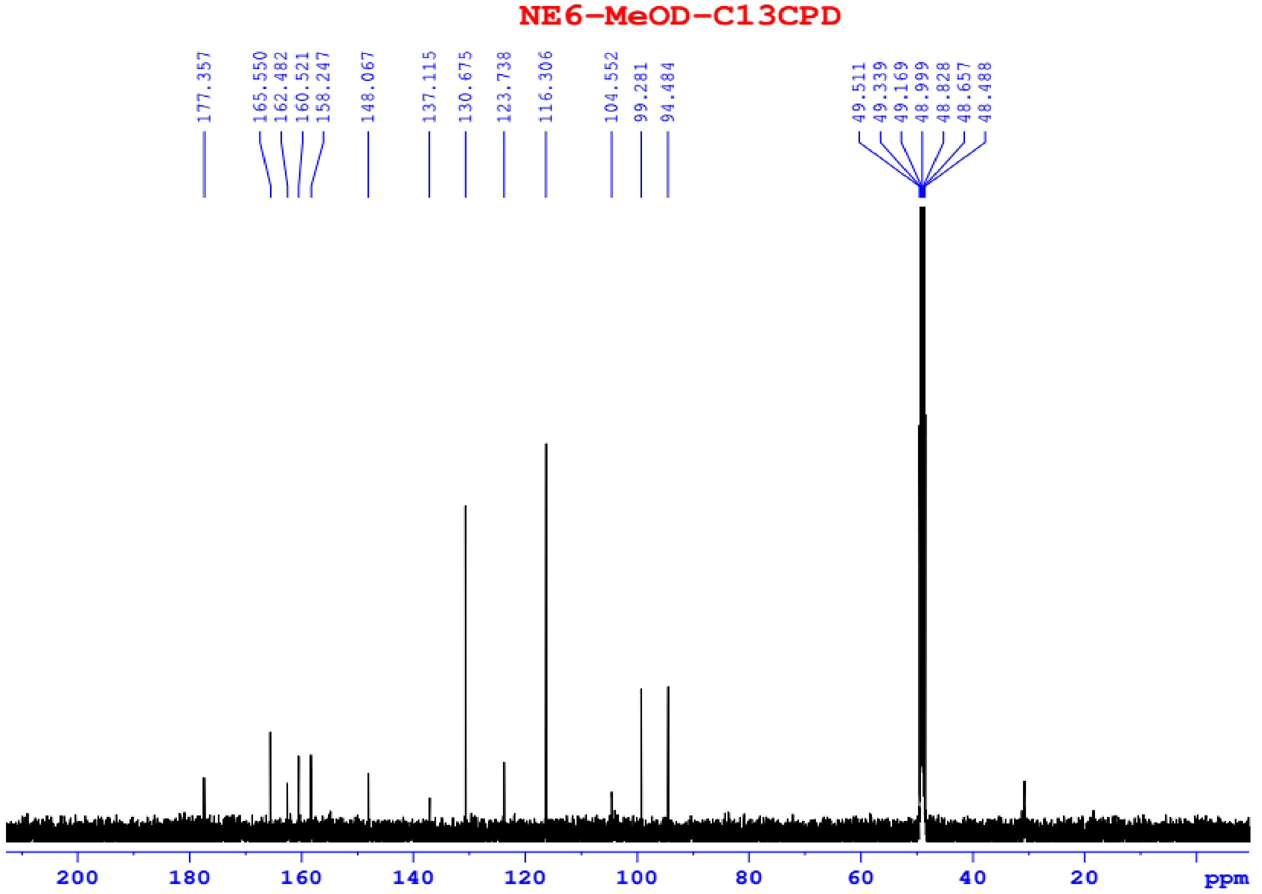

Figure S9. ^{13}C-NMR spectrum of compound **5**

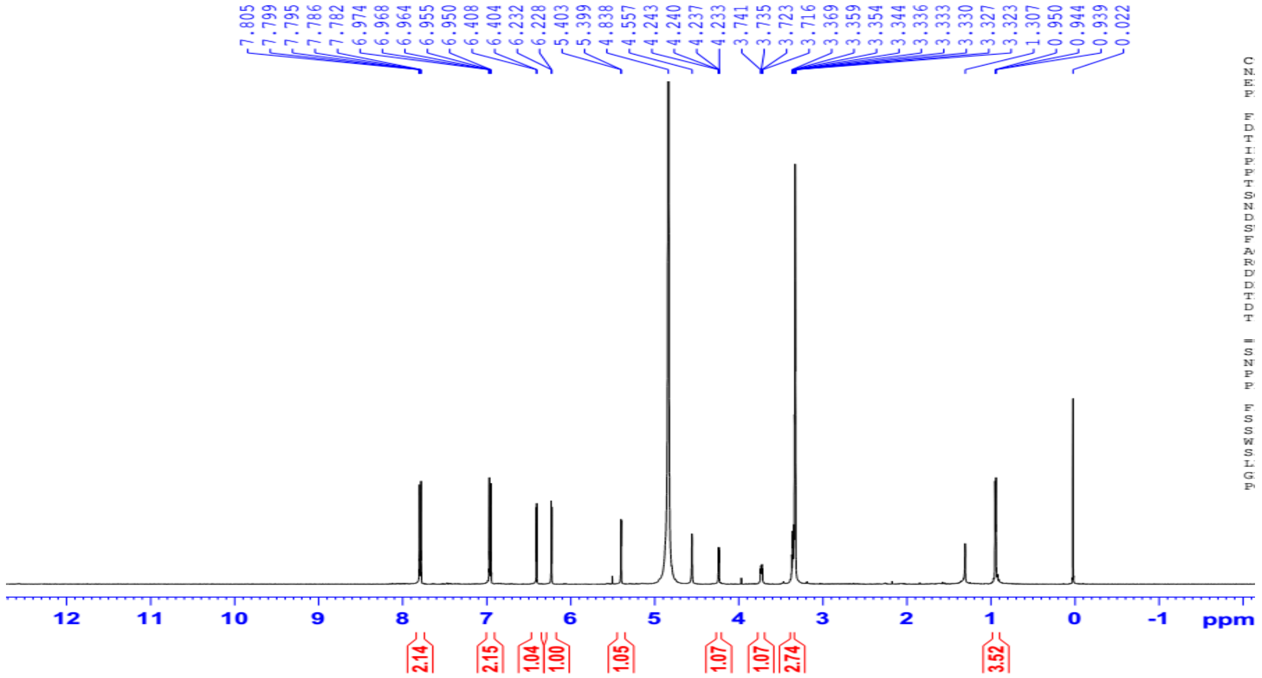

Figure S10. ^{1}H-NMR spectrum of compound **6**

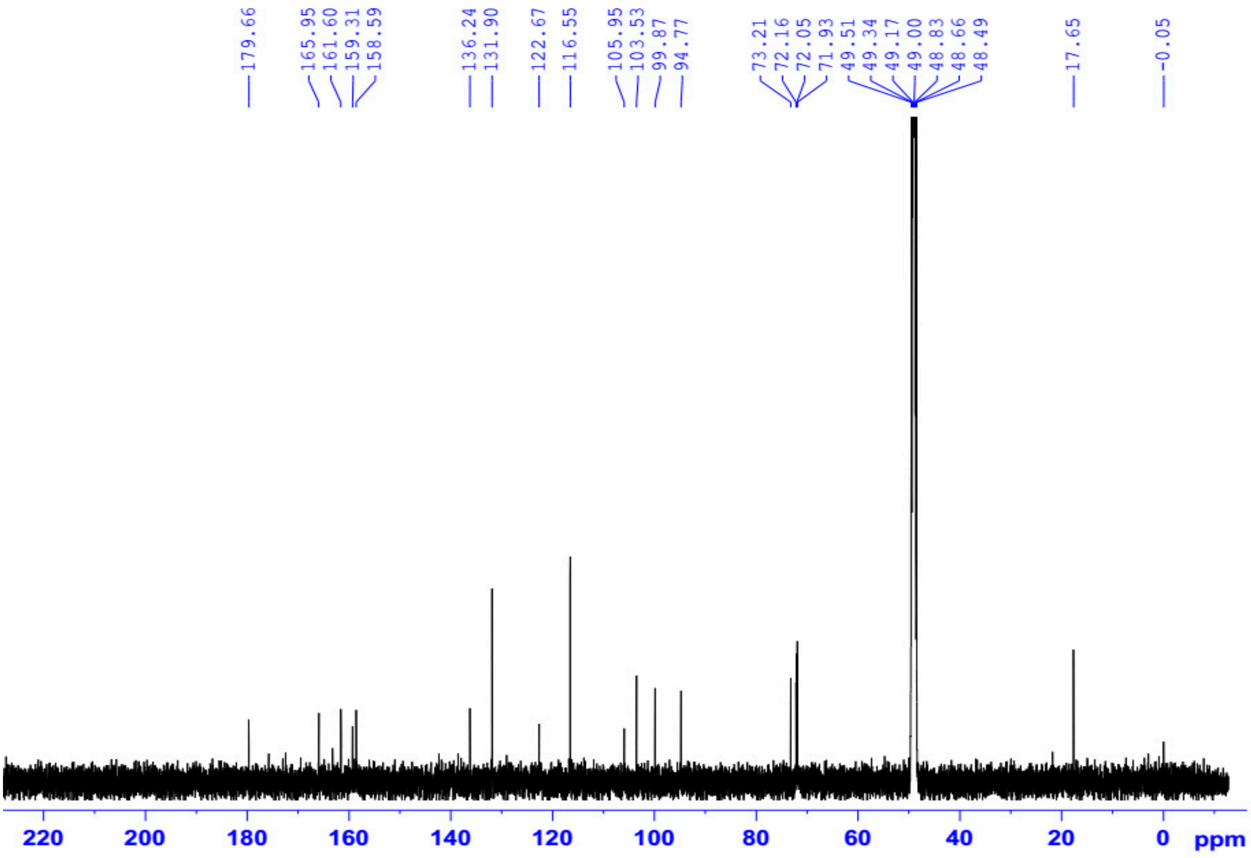

Figure S11. ^{13}C-NMR spectrum of compound **6**

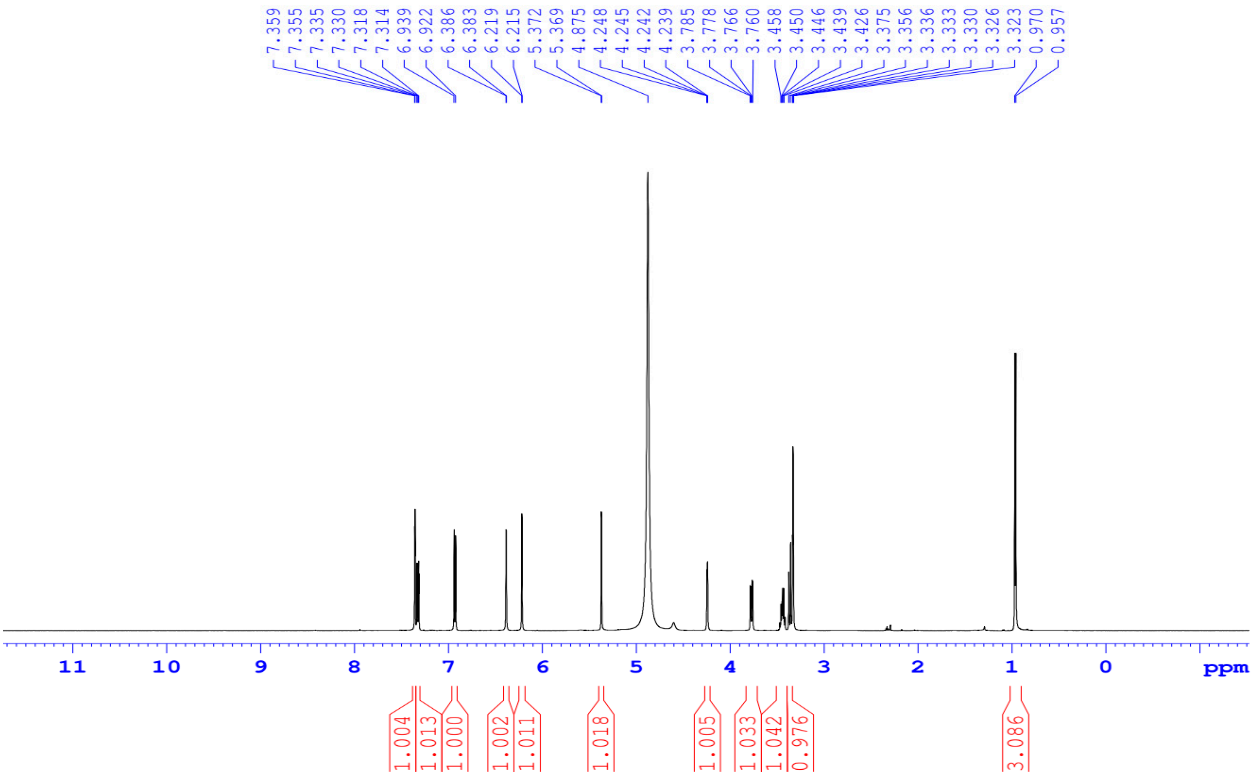

Figure S12. ^{1}H-NMR spectrum of compound **7**

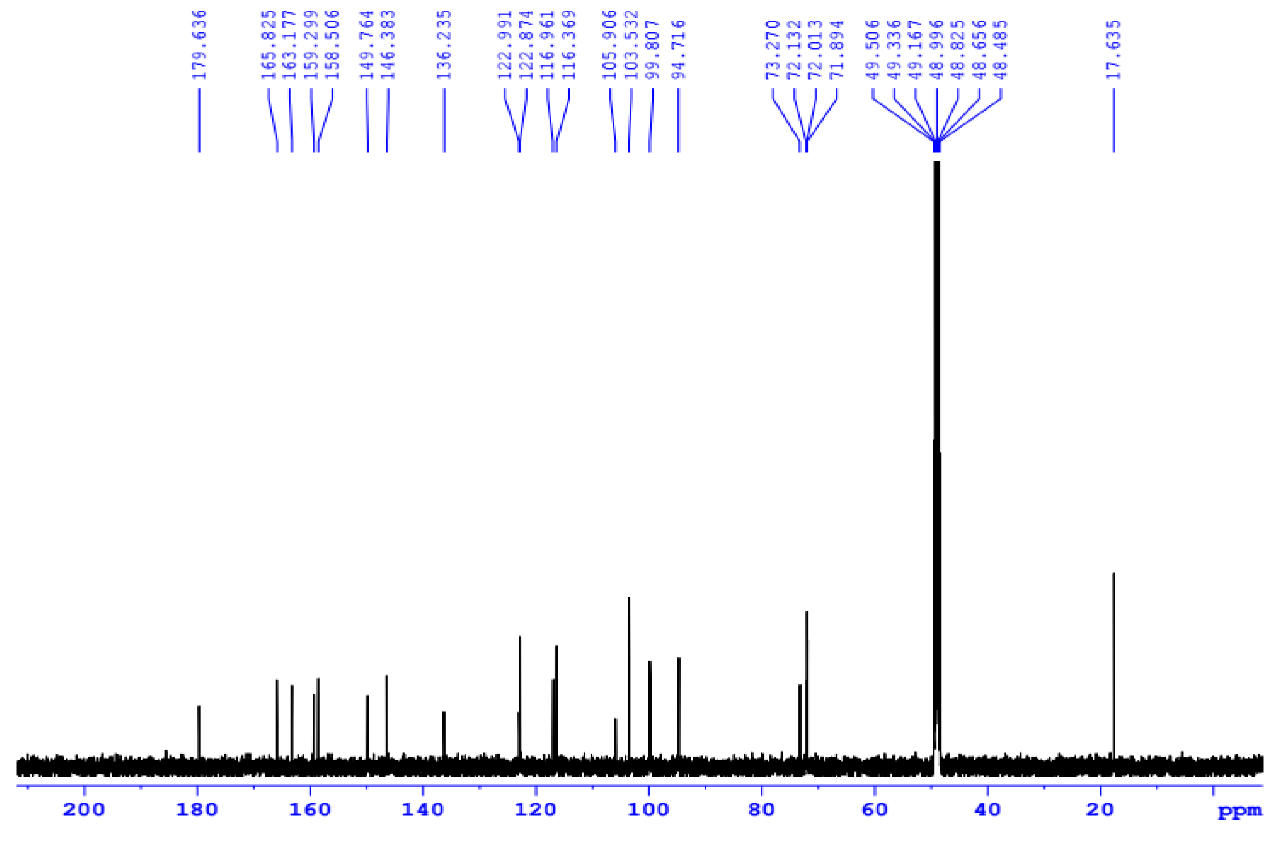

Figure S13. ^{13}C-NMR spectrum of compound **7**

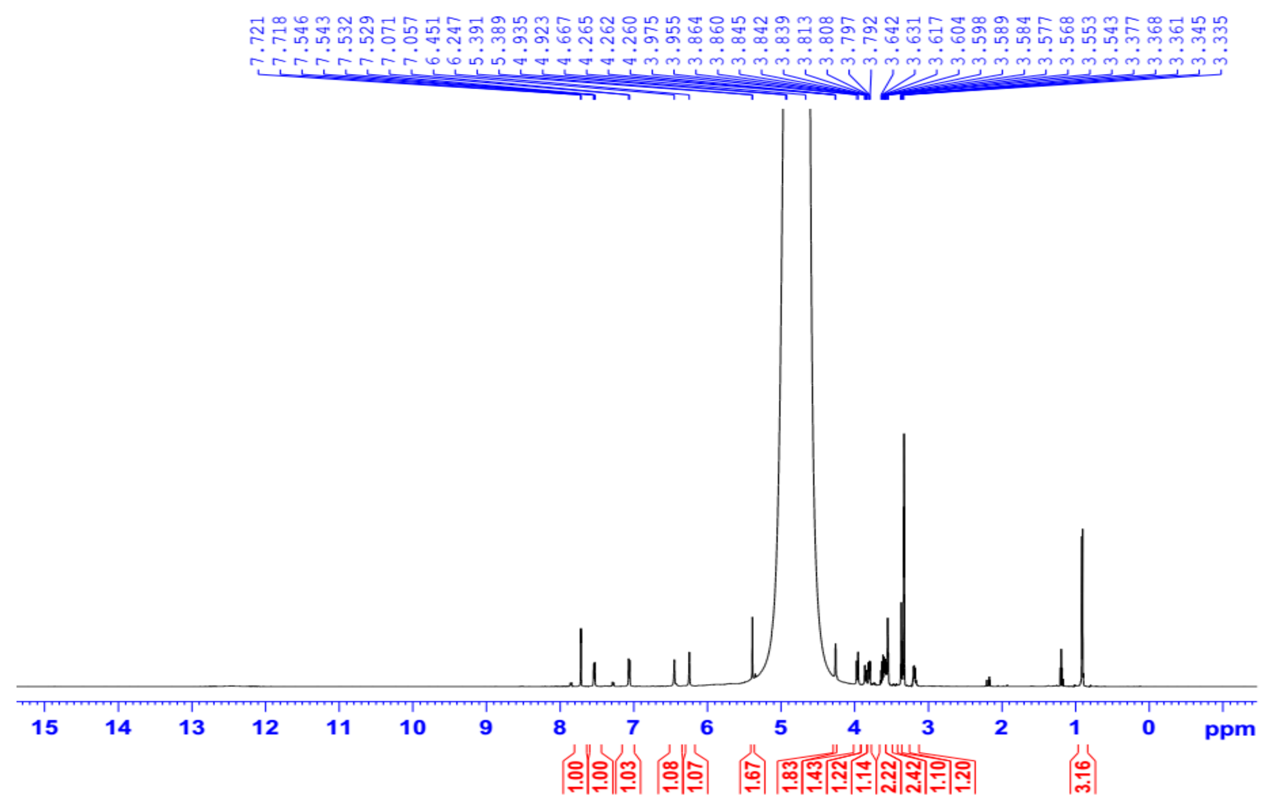

Figure S14. ^{1}H-NMR spectrum of compound **8**

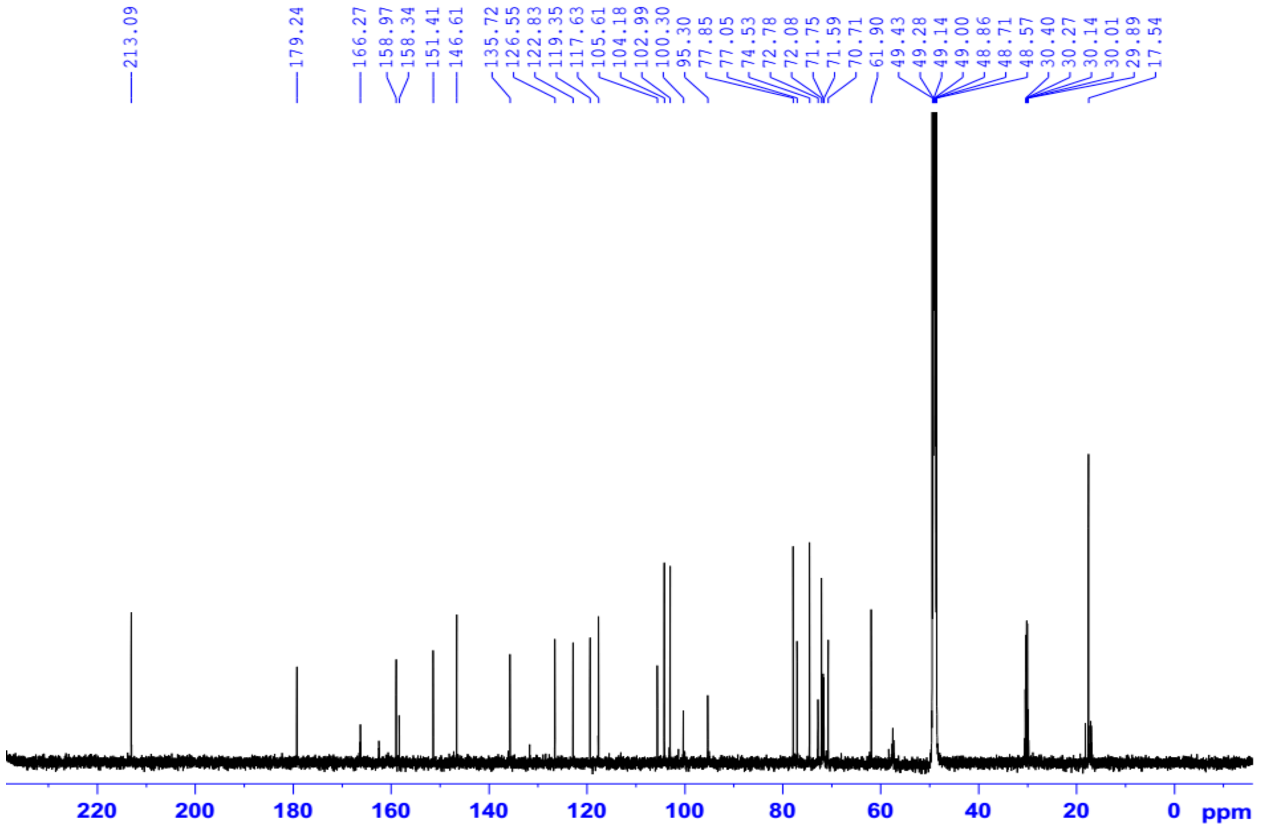

Figure S15. ^{13}C-NMR spectrum of compound **8**

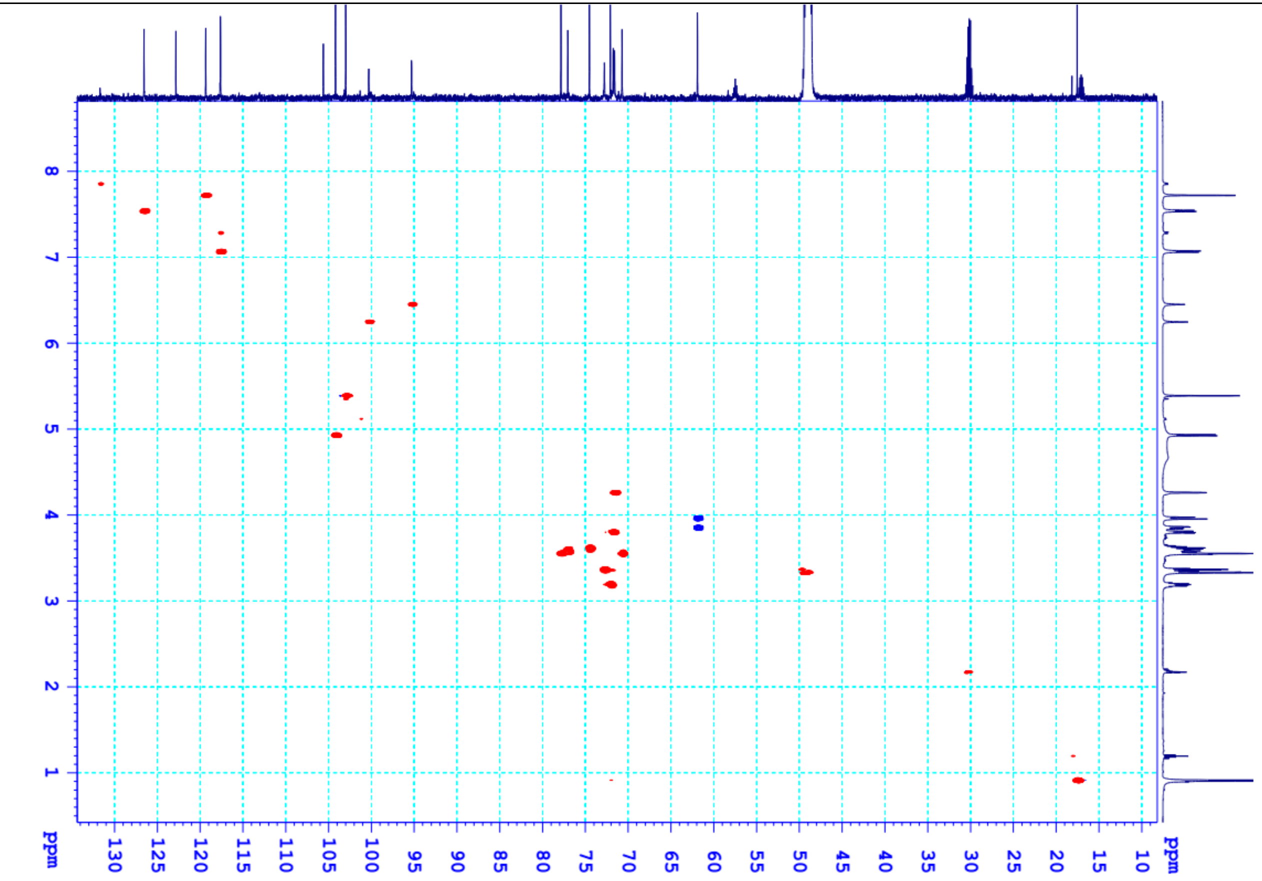

Figure S16. HSQC spectrum of compound **8**

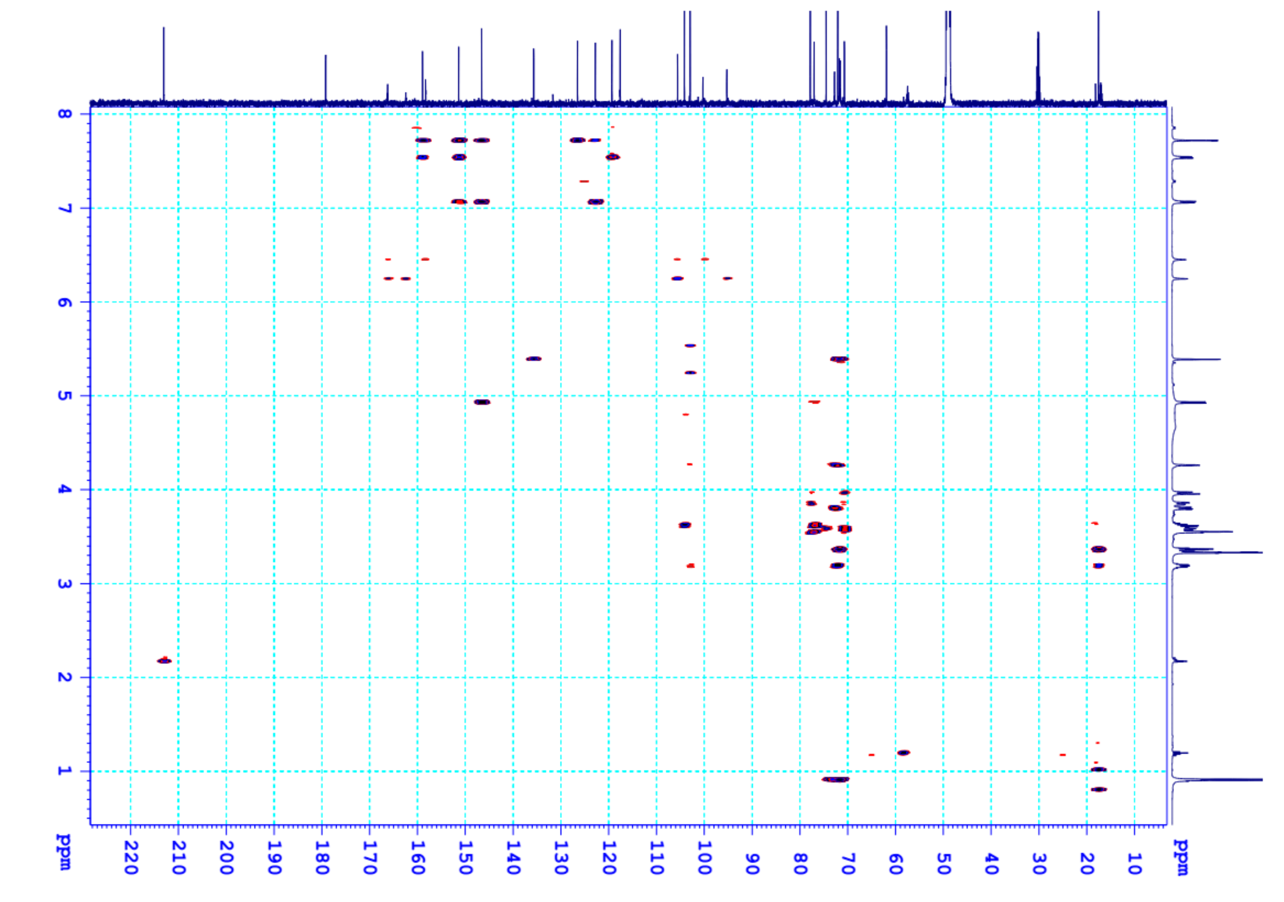

Figure S17. HMBC spectrum of compound **8**

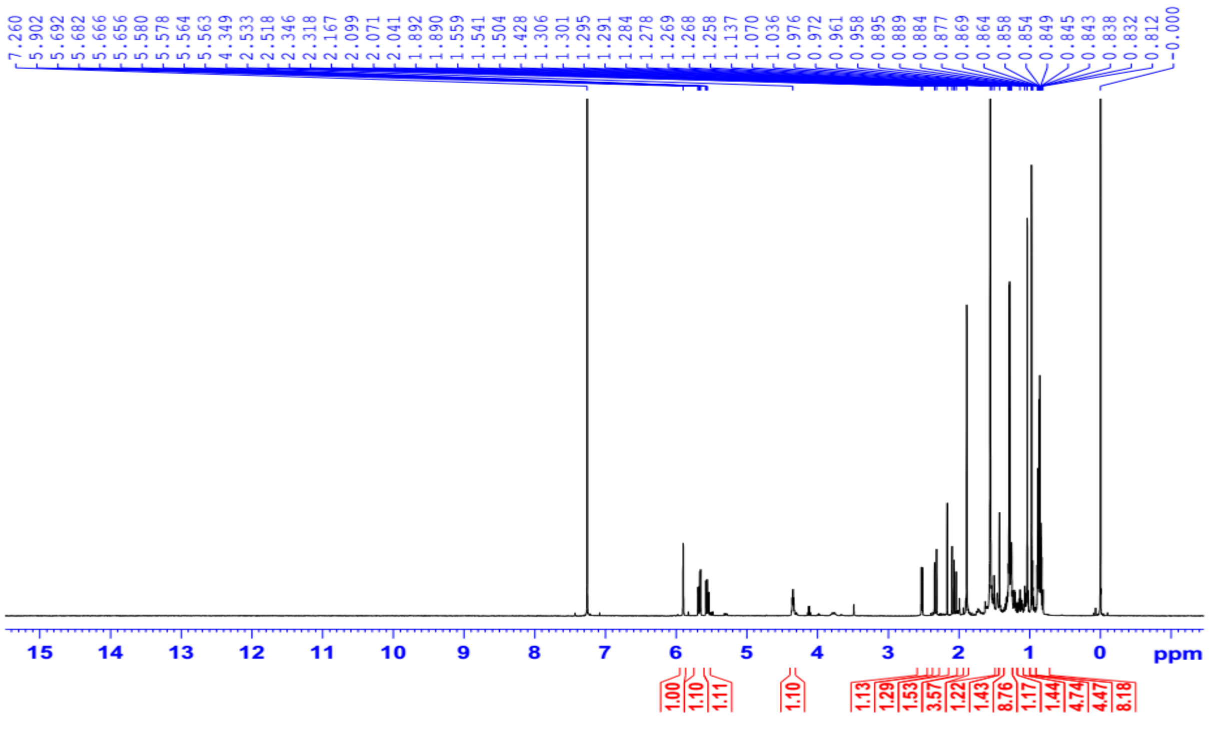

Figure S18. ^{1}H-NMR spectrum of compound **9**

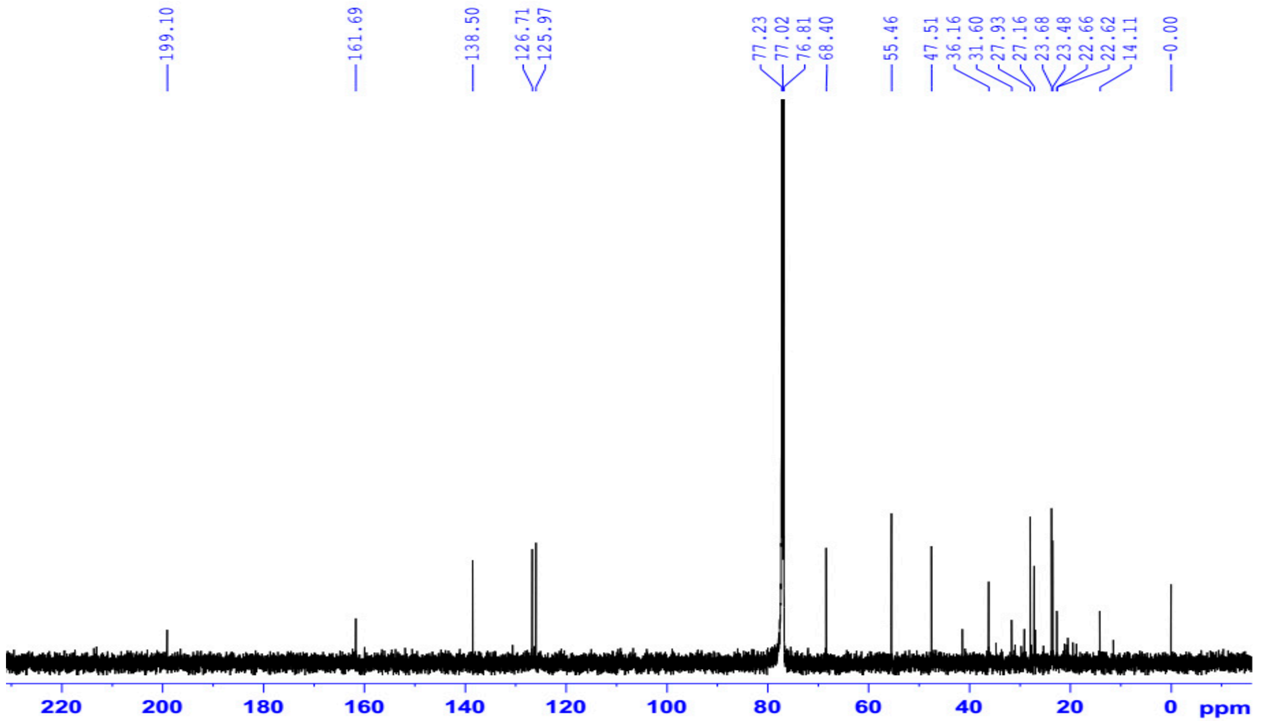

Figure S19. ^{13}C-NMR spectrum of compound **9**

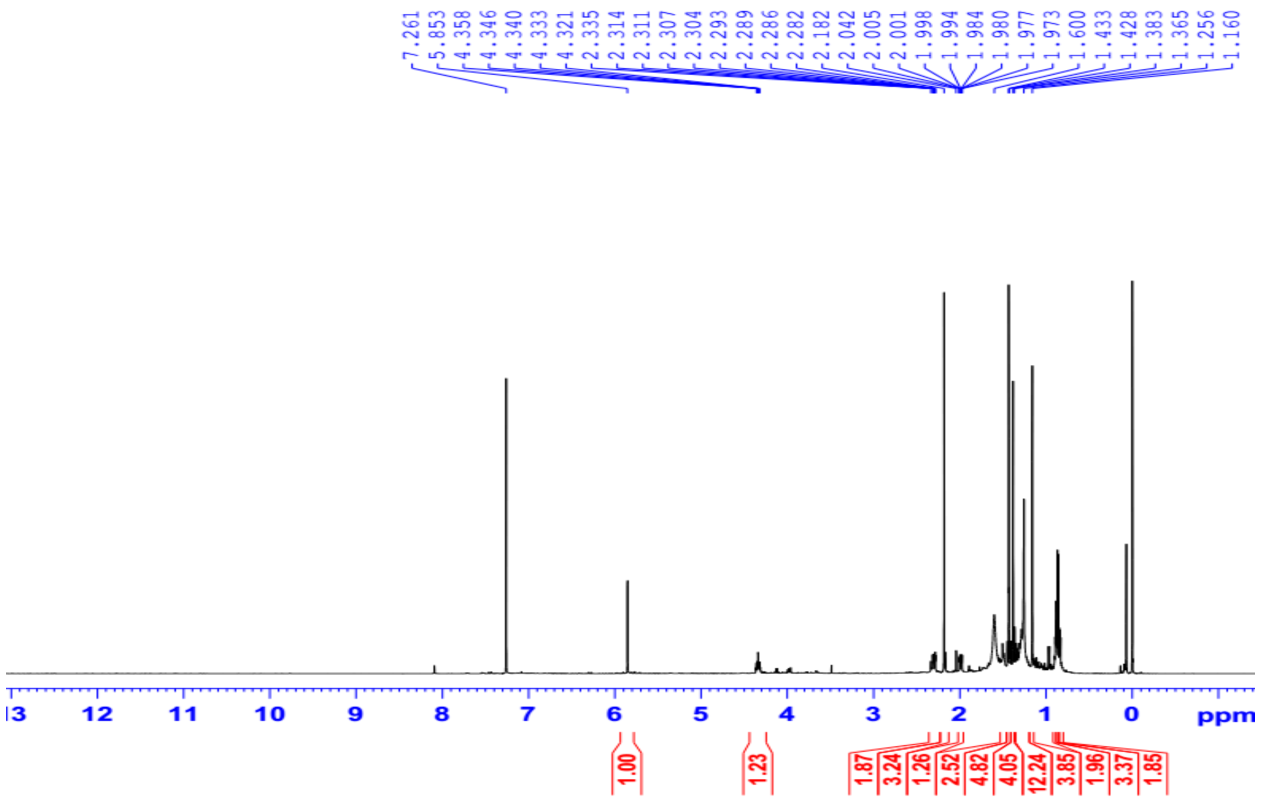

Figure S20. ^{1}H-NMR spectrum of compound **10**

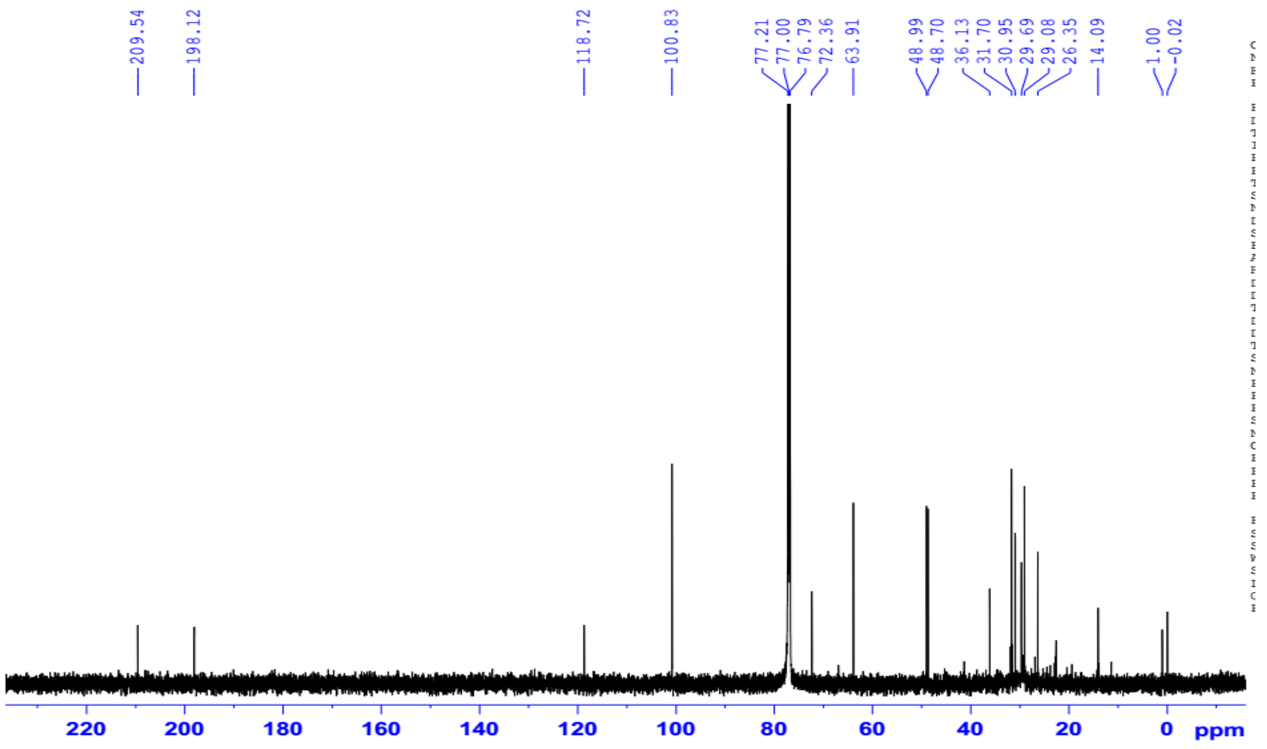

Figure S21. ^{13}C-NMR spectrum of compound **10**

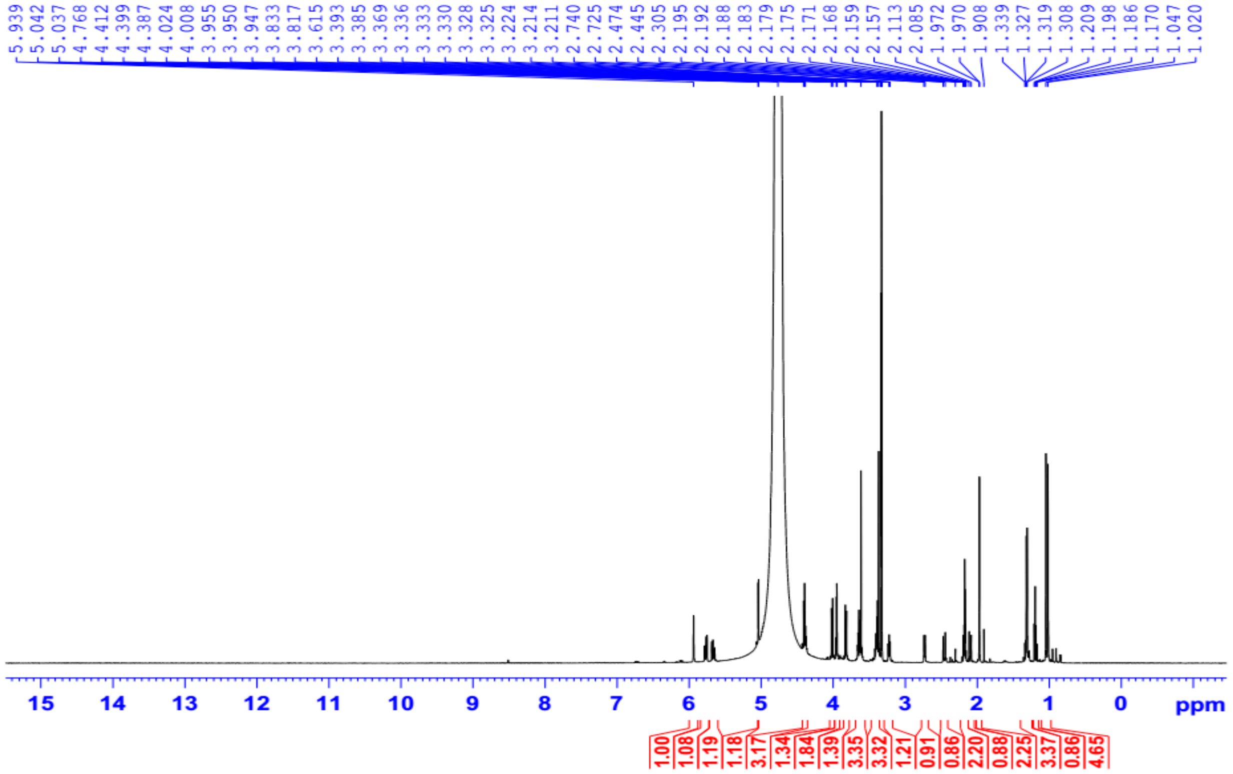

Figure S22. ^{1}H-NMR spectrum of compound **11**

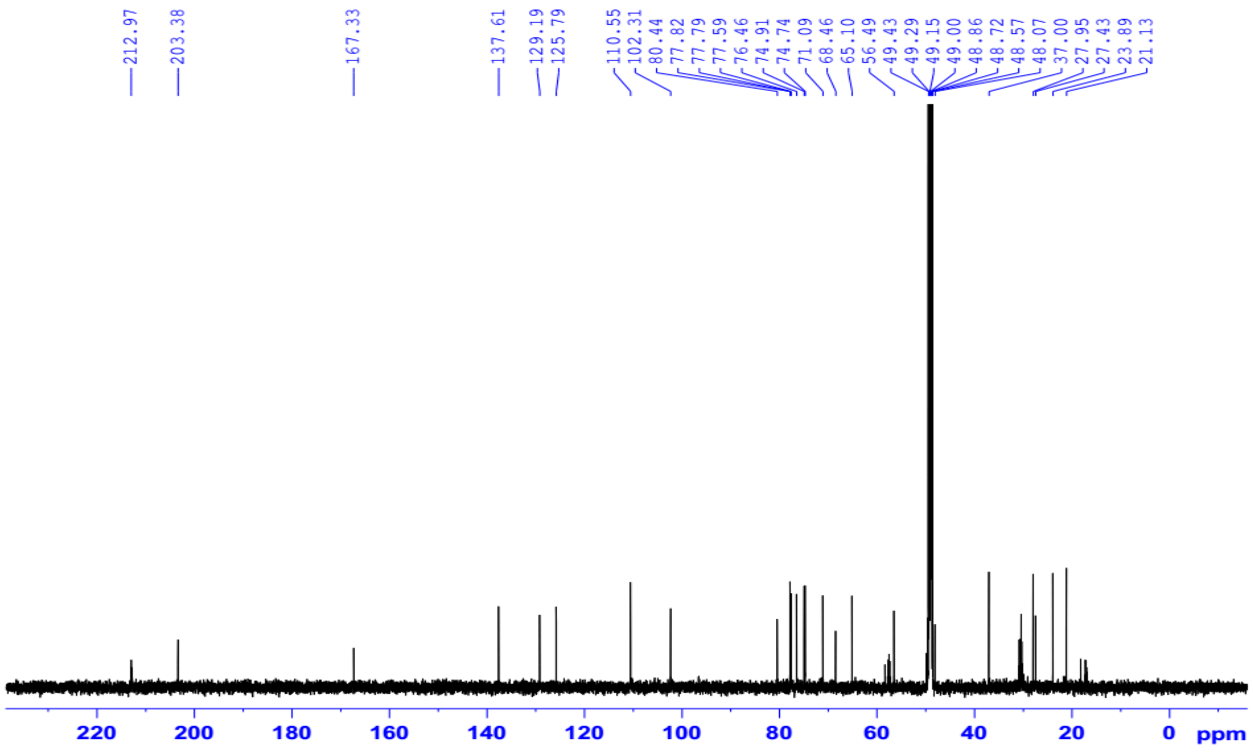

Figure S23. ^{13}C-NMR spectrum of compound **11**

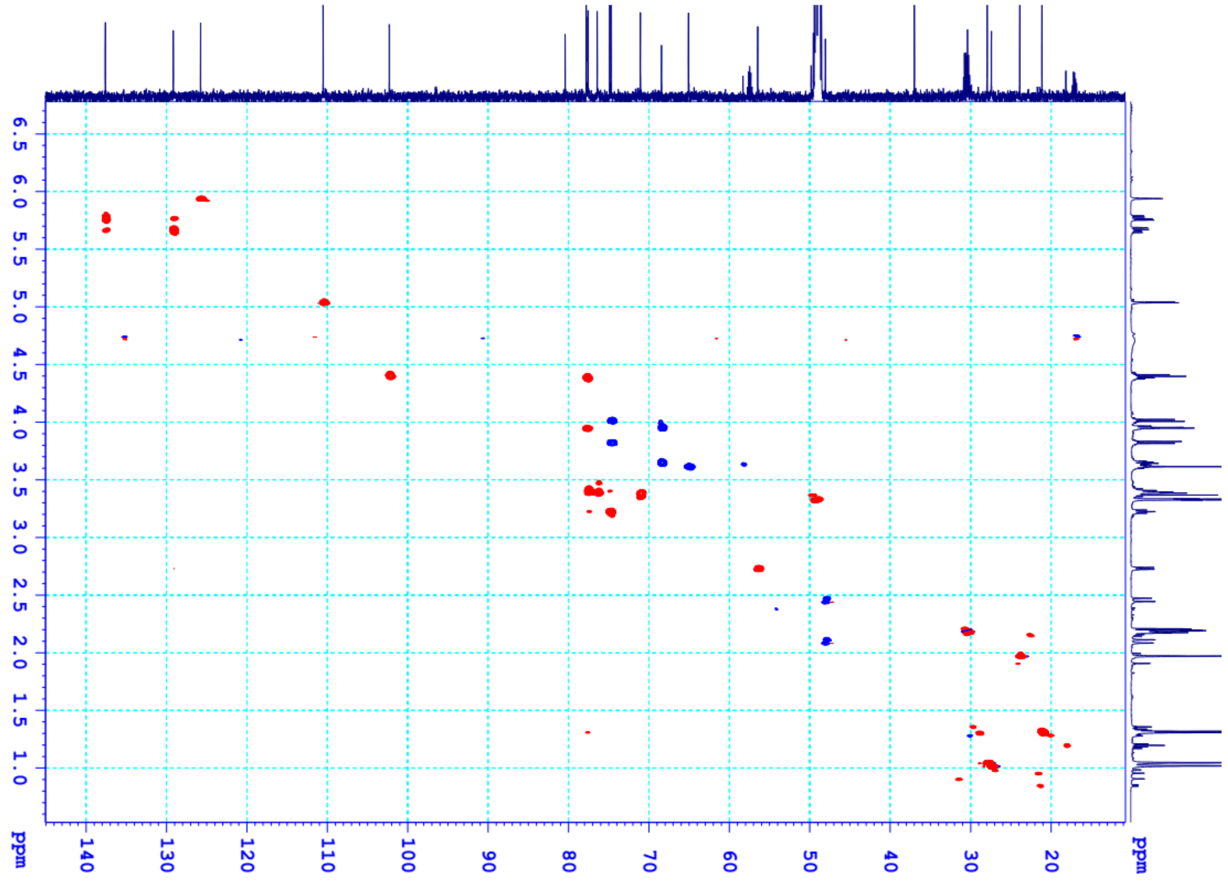

Figure S24. HSQC spectrum of compound **11**

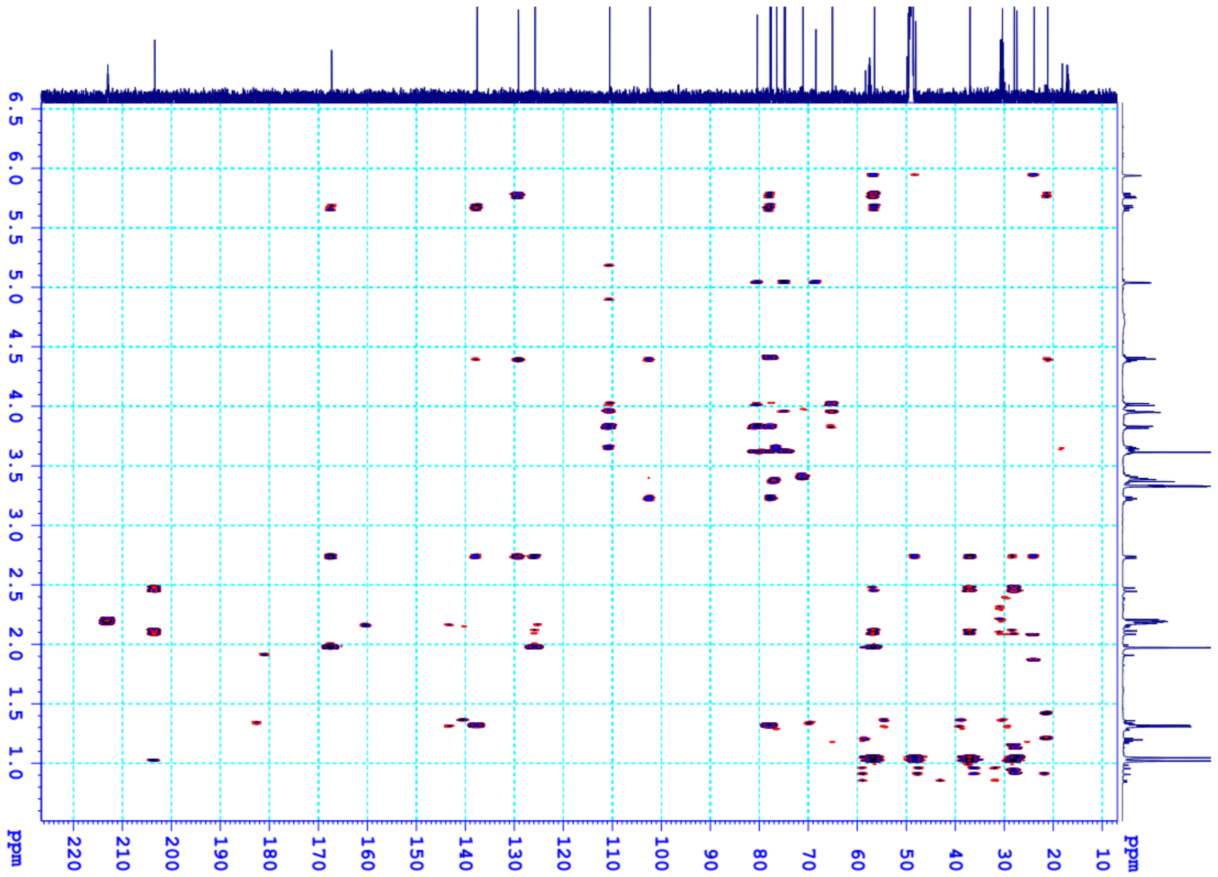

Figure S25. HMBC spectrum of compound **11**

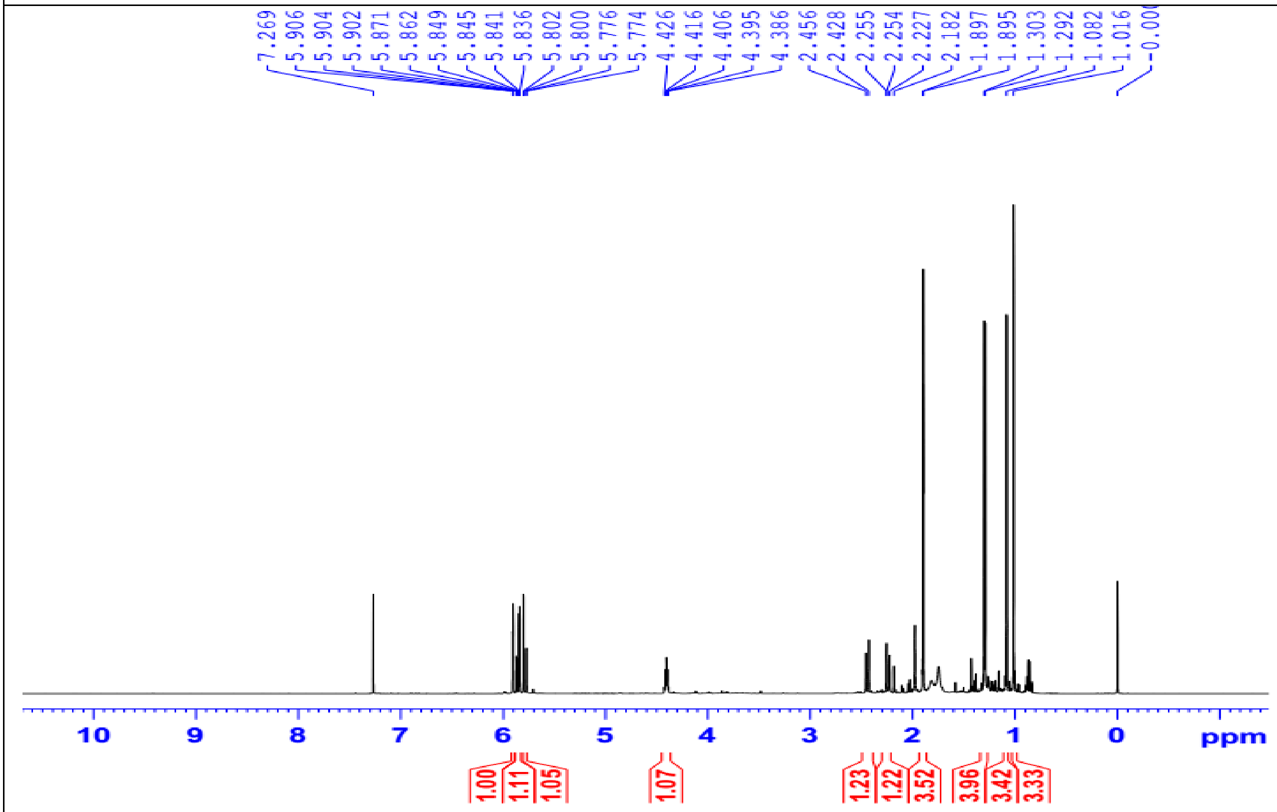

Figure S26. ^{1}H-NMR spectrum of compound **12**

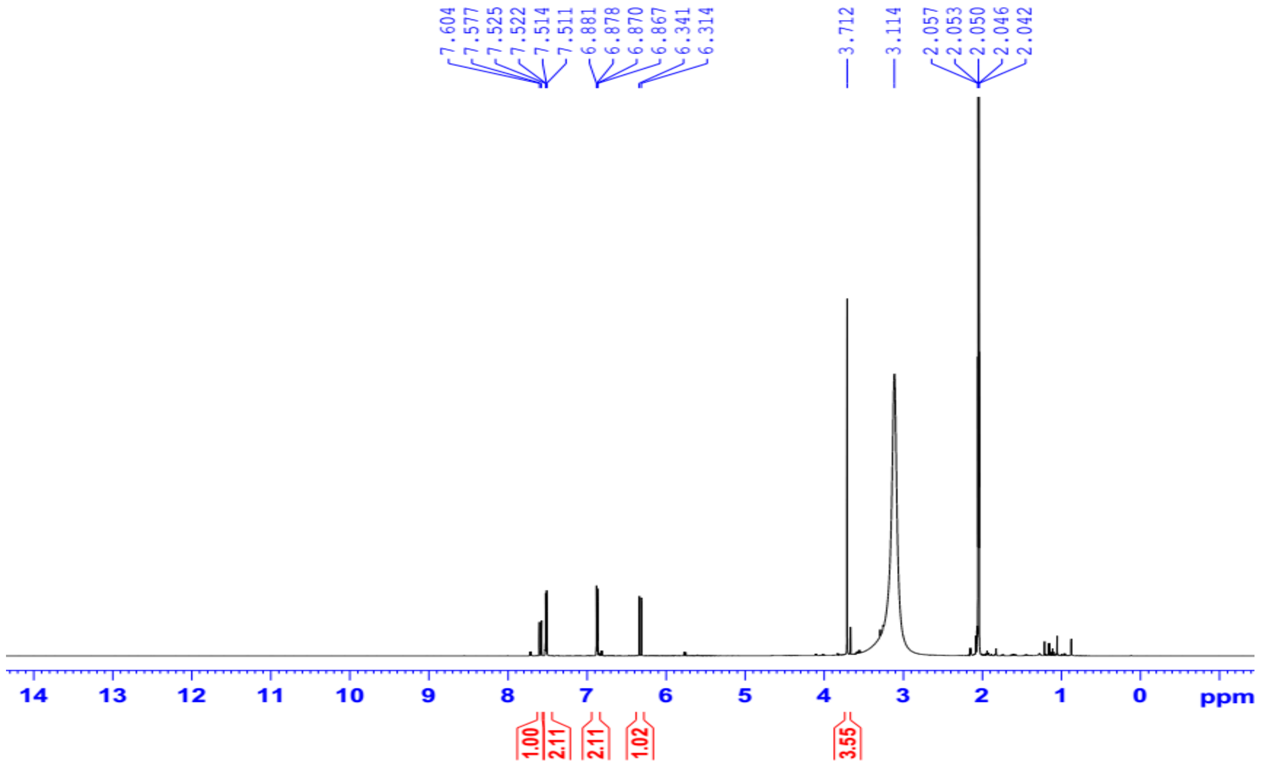

Figure S27. ^{1}H-NMR spectrum of compound **13**

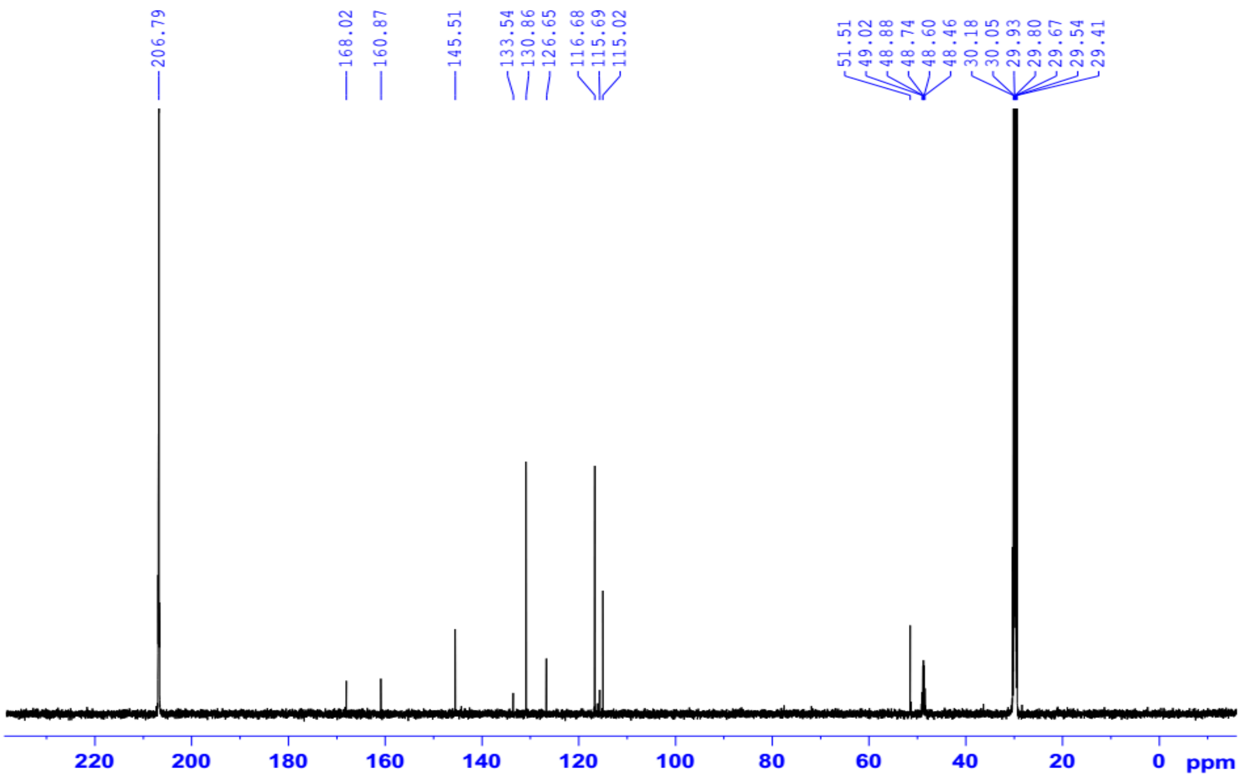

Figure S28. ^{13}C-NMR spectrum of compound **13**

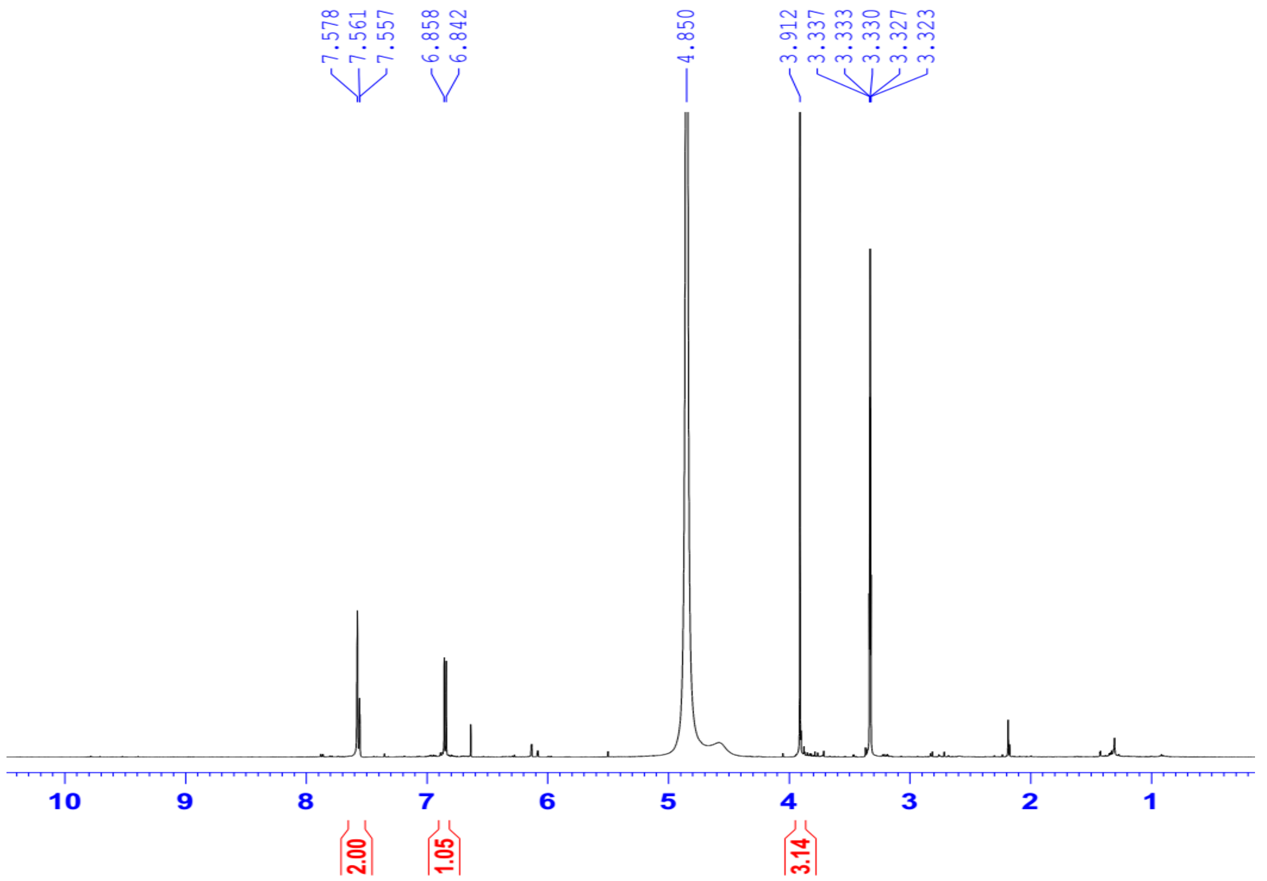

Figure S29. ^{1}H-NMR spectrum of compound **14**

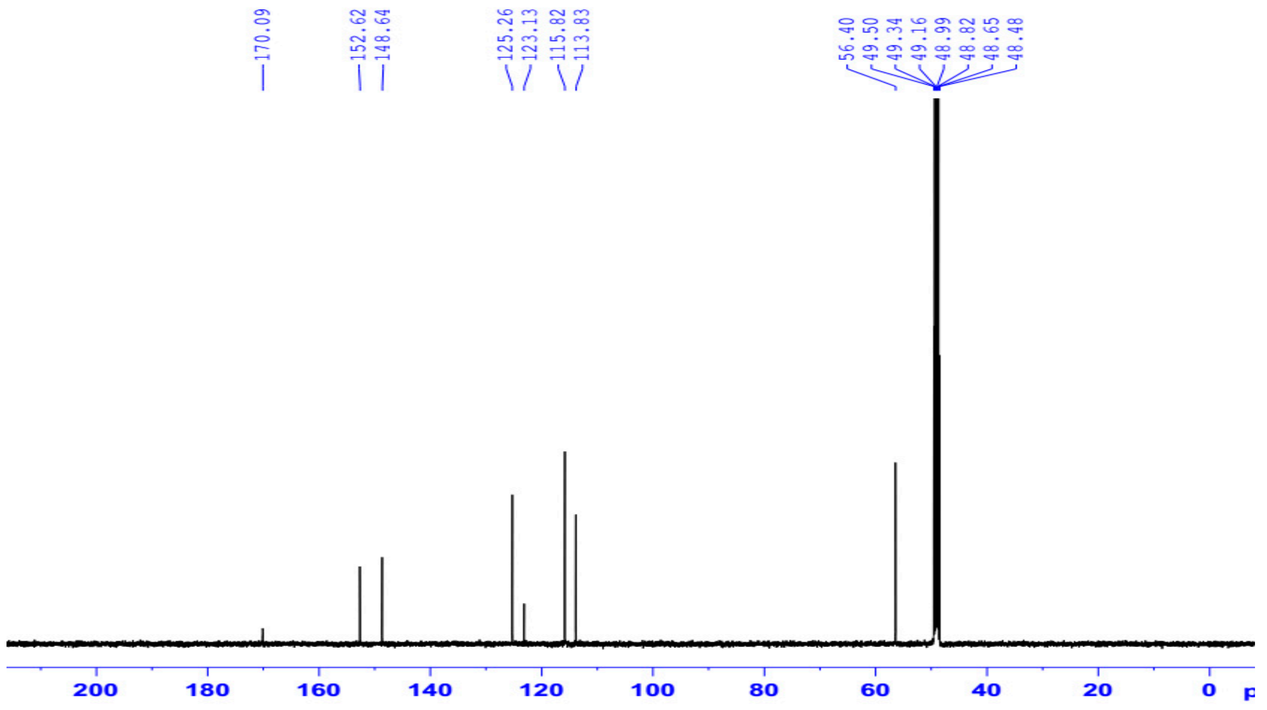

Figure S30. ^{13}C-NMR spectrum of compound **14**

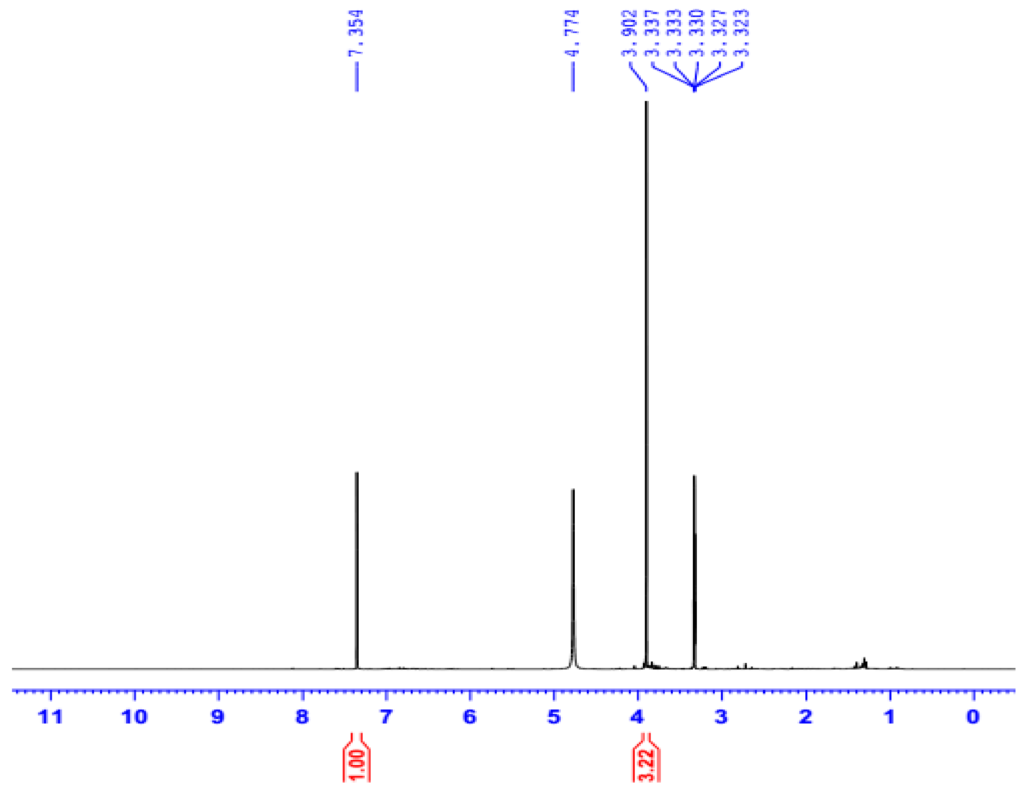

Figure S31. ^{1}H-NMR spectrum of compound **15**

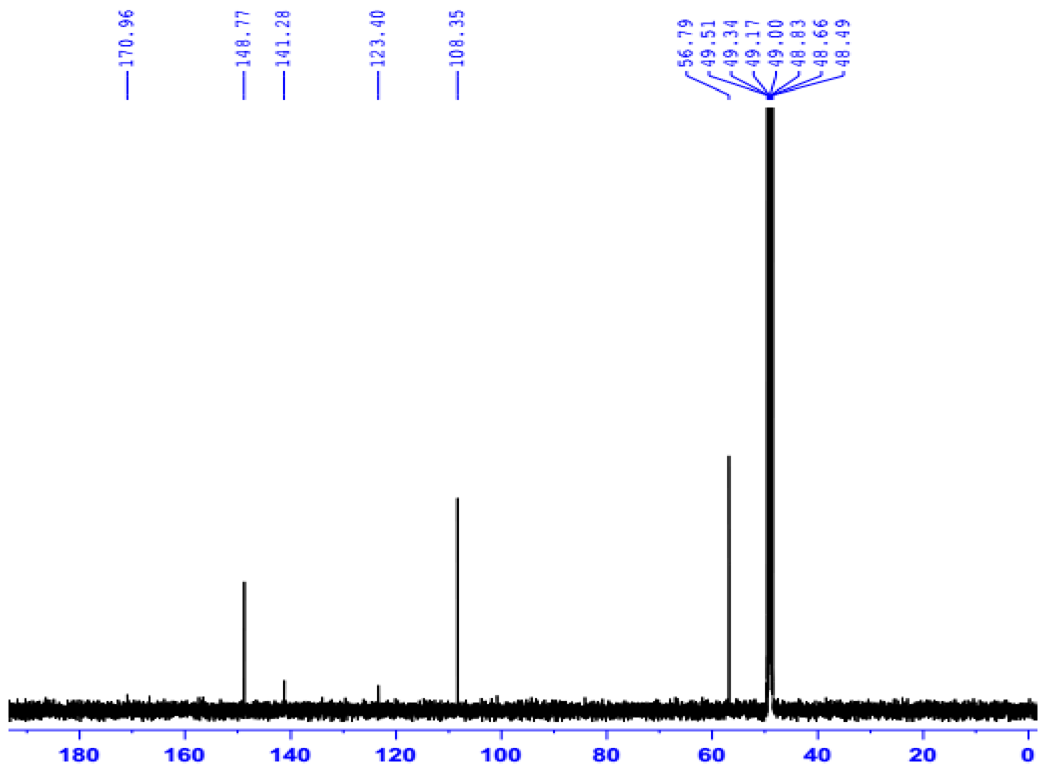

Figure S32. ^{13}C-NMR spectrum of compound **15**

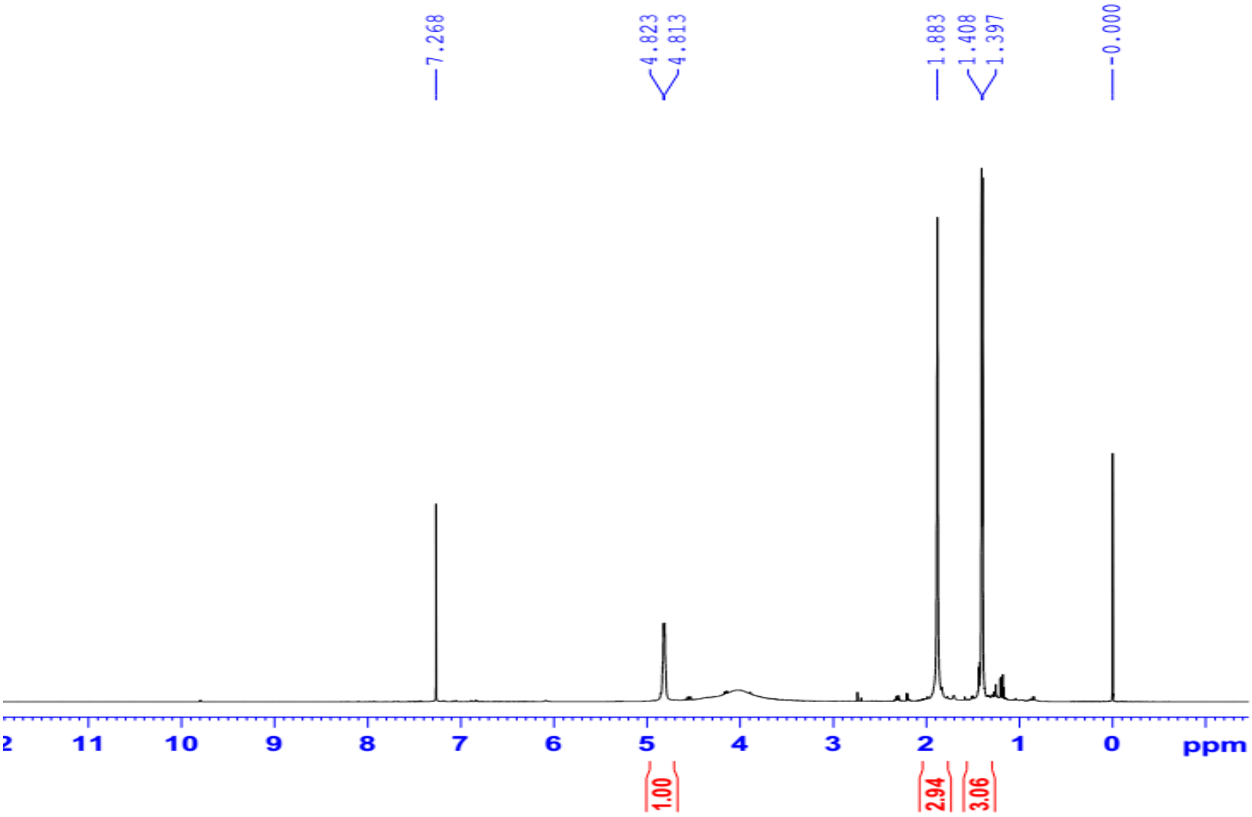

Figure S33. ^{1}H-NMR spectrum of compound **16**

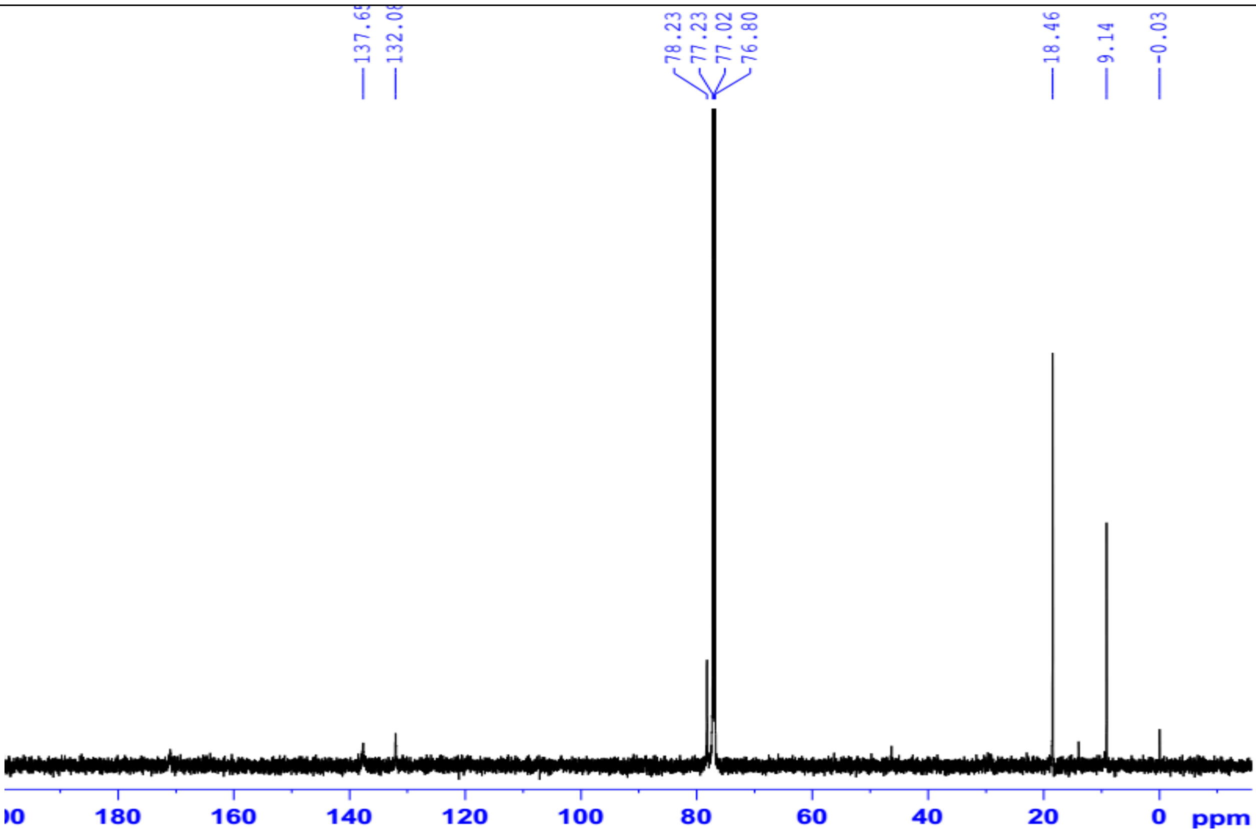

Figure S34. ^{13}C-NMR spectrum of compound **16**

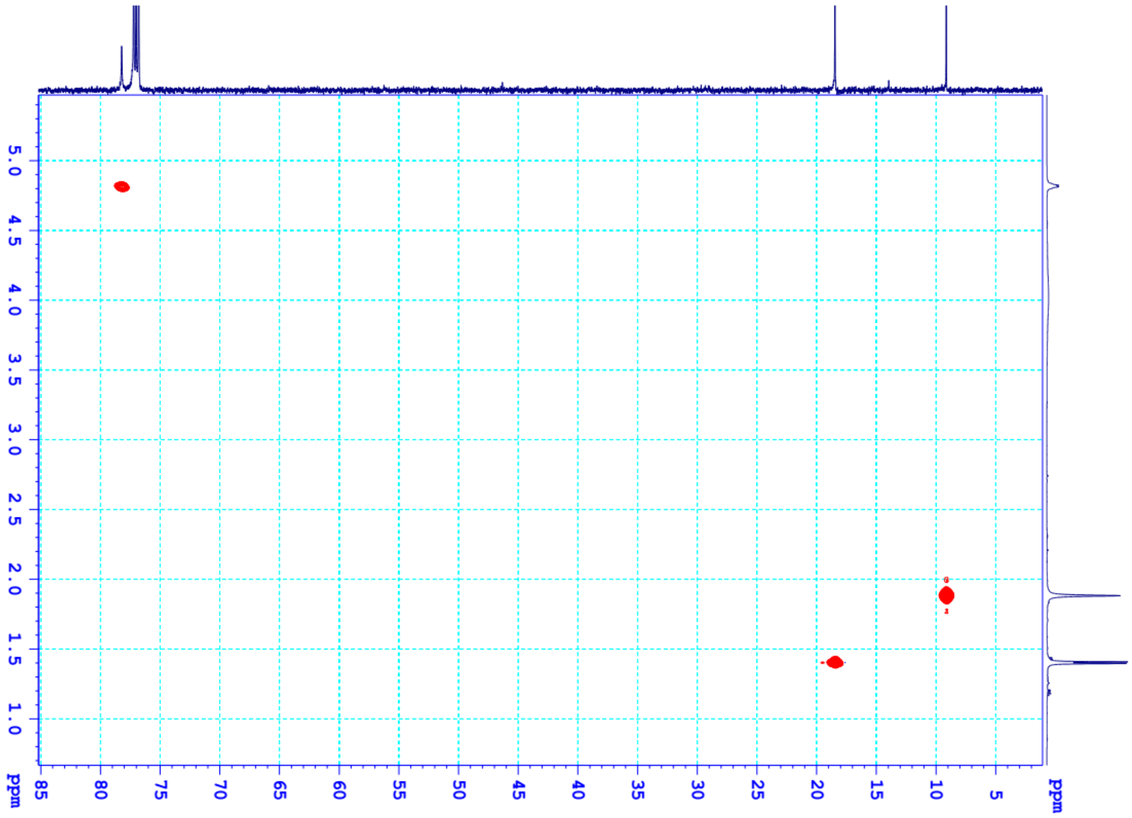

Figure S35. HSQC spectrum of compound **16**

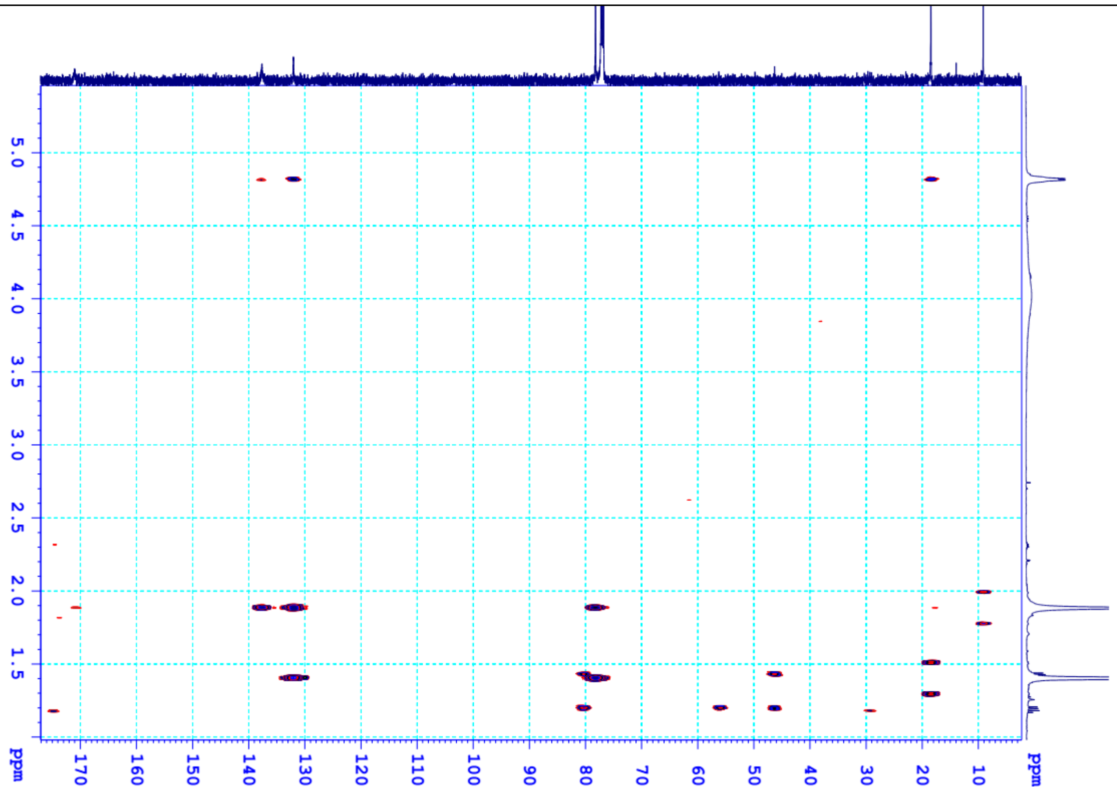

Figure S36. HMBC spectrum of compound **16**
